# Supplementary material for: Development of a versatile nuclease prime editor with upgraded precision
Source: Nat Commun. 2023 Jan 19;14:305. doi: 10.1038/s41467-023-35870-0 (PMC9852468; doi:10.1038/s41467-023-35870-0)

Supplementary information

Development of a versatile nuclease prime editor with upgraded precision

Contents:

Supplementary Figures

Supplementary Figure 1. DNA-PK inhibitor (NU7441) promoted PE-nuclease-dependent insertional editing.

Supplementary Figure 2. Selected 53BP1-targeting ubiquitin variants enhanced PEn-mediated genome editing.

Supplementary Figure 3. 53BP1-targeting ubiquitin variants decreased PEn-associated imprecise edits to improve the purities within RT-driven edits.

Supplementary Figure 4. The purity of prime editing induced by uPEn system.

Supplementary Figure 5. The editing efficiencies of PEn/uPEn compared to the Cas9/template and PE2max system.

Supplementary Figure 6. Examination of cell viability in response to the transfected i53 or uPEn.

Supplementary Figure 7. The potential mechanism(s) underlying PEn- and uPEn-dependent editing.

Supplementary Figure 8. CRISPResso2-based analyses of editing outcomes by PEn/uPEn at *SEC61B.*

Supplementary Figure 9. CRISPResso2-based analyses of editing outcomes by PEn (± NU7441) with HR-free pegRNAs.

Supplementary Figure 10. Targeted sequence insertion, deletion and replacement by uPEn system in U2OS cells.

Supplementary Figure 11. Statistical analysis of prime editing efficiency and indels in HEK293 and U2OS cells.

Supplementary Figure 12. Comparisons of uPEn and PE5max for installing base conversions in HeLa cells.

Supplementary Figure 13. Comparisons among uPEn, PE3max and configuration-simplified PE5max for base conversions at previously tested sites.

Supplementary Figure 14. Benchmarking the performances of uPEn for insertion, deletion and replacement.

Supplementary Figure 15. Comparison of potential off-target rates for PEn and uPEn at three genomic sites in HEK293T cells.

Supplementary Figure 16. Whole genome sequencing analyses of off-target effects associated with PE5max, PEn and uPEn.

Supplementary Figure 17. Flow cytometry gating strategies.

Supplementary Tables

Supplementary Table 1. Primers used for constructing of pegRNA and HR-pegRNA plasmids.

Supplementary Table 2. Primers used for ssDNA donors.

Supplementary Table 3. Primers used for pegRNA RT-qPCR analysis.

Supplementary Notes

Supplementary Note 1. The sequence of Blank Control (mCherry).

Supplementary Note 2. The sequence of mCherry-P2A-Ub (WT).

Supplementary Note 3. The sequence of mCherry-P2A-H04.

Supplementary Note 4. The sequence of mCherry-P2A-A10.

Supplementary Note 5. The sequence of mCherry-P2A-A11.

Supplementary Note 6. The sequence of mCherry-P2A-C08.

Supplementary Note 7. The sequence of mCherry-P2A-G08.

Supplementary Note 8. The sequence of mCherry-P2A-G08(I44A).

Supplementary Note 9. The sequence of PEn.

Supplementary Note 10. The sequence of uPEn2.

Supplementary Note 11. The sequence of uPEn3.

Original scanned blots for Western results presented in the supplementary figures

Supplementary Figures

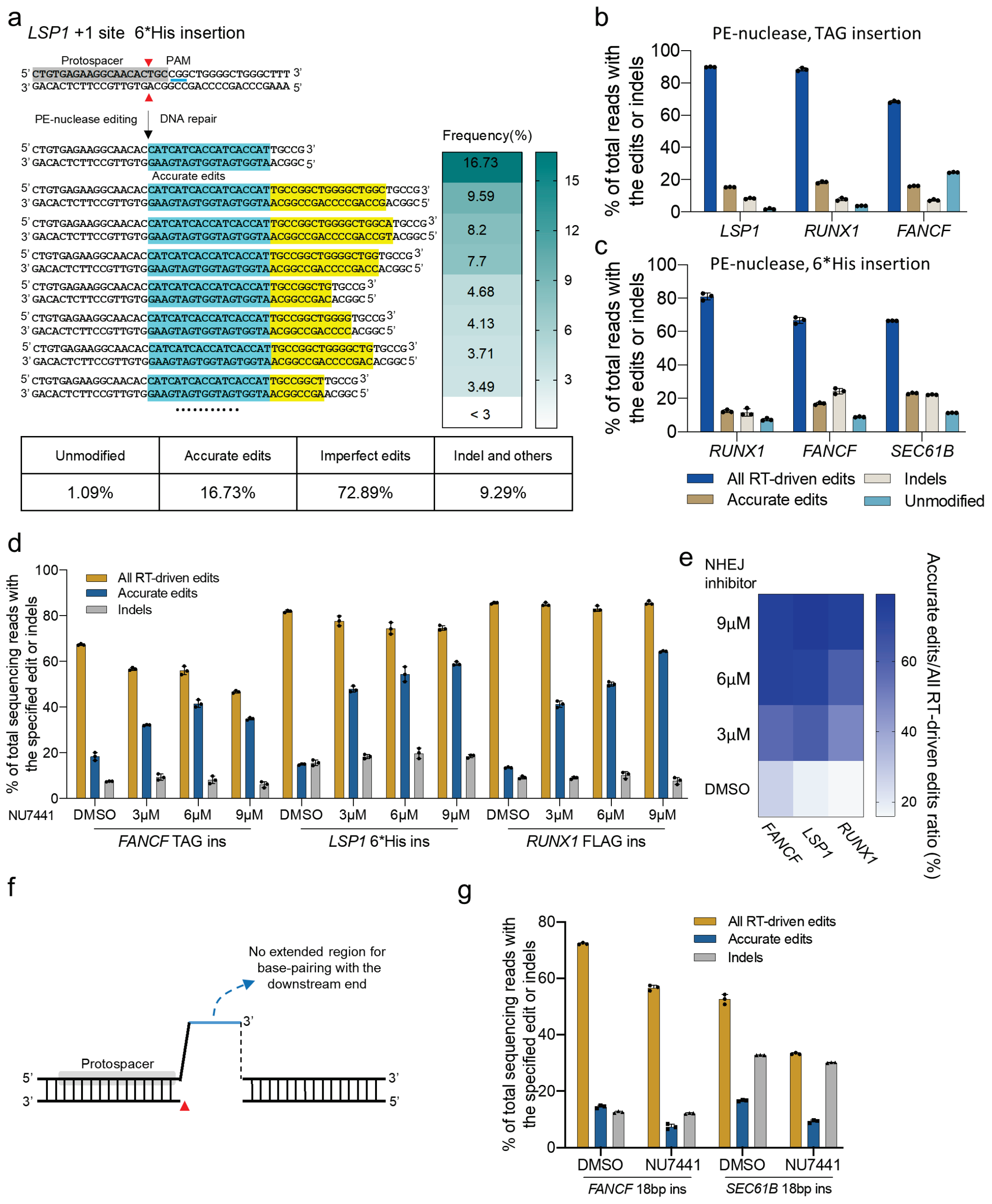

Supplementary Figure 1. DNA-PK inhibitor (NU7441) promoted PE-nuclease-dependent insertional editing.

a. An example of editing outcomes of PE-nuclease-induced 6*His insertion at *LSP1* site in HEK293T cells. Gray fill indicates the spacer region of pegRNA. Red arrows indicate the position of pegRNA-induced DSB. In the lists of editing outcomes, the blue fills indicate the designed insertion sequence. The yellow fills indicate the unwanted, extra inserted sequences derived from the 5′ portion of RTT. The

frequencies corresponding to these genotypes are showed on the right, and covered by green shades mapping the levels. The summary for the percentage corresponding to each type of edits is shown on the bottom.

b, c. Quantitation of editing outcomes by PE-nuclease for targeted TAG (**b**) or 6*His (**c**) insertions at three different loci in HEK 293T cells. All RT-driven edits consist of desired edits and imprecise edits containing RTT-derived sequences. Accurate edits represent desired edits. Values and error bars reflect the means and s.d. of three biological replicates.

d. Prime editing outcomes of PE-nuclease in HEK293T cells treated with DMSO or indicated doses of NU7441. The PE-nuclease was programmed for targeted insertions of TAG, 6*His and FLAG at the gene loci of *FANCF*, *LSP1*, and *RUNX1*, respectively. Values and error bars reflect the means and s.d. of three biological replicates. The inhibitor treatment clearly increased the levels of precise edits (blue bars).

e. The ratio of accurate edits relative to all RT-driven edits in (**d**) were determined. The darker color indicates higher percentage of accurate edits.

f. The illustration of a specific PEn condition (alignment-relaxed) where the RT-dependent 3′ overhang lacked the segment of homology to the downstream DSB end. The designed sequence for insertion is marked in blue within the overhang.

g. Editing outcomes of the alignment-relaxed PEn (**f**) at the *FANCF* and *SEC61B* sites. The cells were treated with DMSO or NU7441 (9 μM). Values and error bars reflect the means and s.d. of three biological replicates.

Source data are provided as a Source Data file.

a

Variants	Amino acid sequence (Red amino acids represent mutated position)
Ub(wt)	MQIFVKTLTGKTITLEVEPSDTIENVKAKIQDKEGIPPDQQRLIFAGKQLEDGRTLSDYNIQKESTLHLVLRLR
A10	MQIYVKTFARKPITLEVEPSDTIENVKAKIQDKEGIPPDQQRLIFAEMRLEDGRTLSDYNIKNDSKLFLVLKNSVT
A11	MLIFVTTDMGMTISLEVEPSDTIENVKAKIQDKEGIPPDQQRLIFGDKDLEDGRTLSDYNIQKESSLNLVLKLR
C08	MQIFVTTDMWMRISLEVEPSDTIENVKAKIQDKEGIPPDQQRLIFGDKDLEDGRTLSDYNIQKESSLNLVLNLR
G08	MLIFVKTLTGKTITLEVEPSDTIENVKAKIQDKEGIPPDQQRLIFAGKSLEDGRTLSDYNILKDSKLHPLLRLR
H04	MRIIVKTFMRKPITLEVEPSDTIENVKAKIQDKEGIPPDQQRLYFAASQLEDGRTLSDYNIQKESTLLLVVRLLRV
G08-I44A	MLIFVKTLTGKTITLEVEPSDTIENVKAKIQDKEGIPPDQQRLAFAGKSLEDGRTLSDYNILKDSKLHPLLRLR

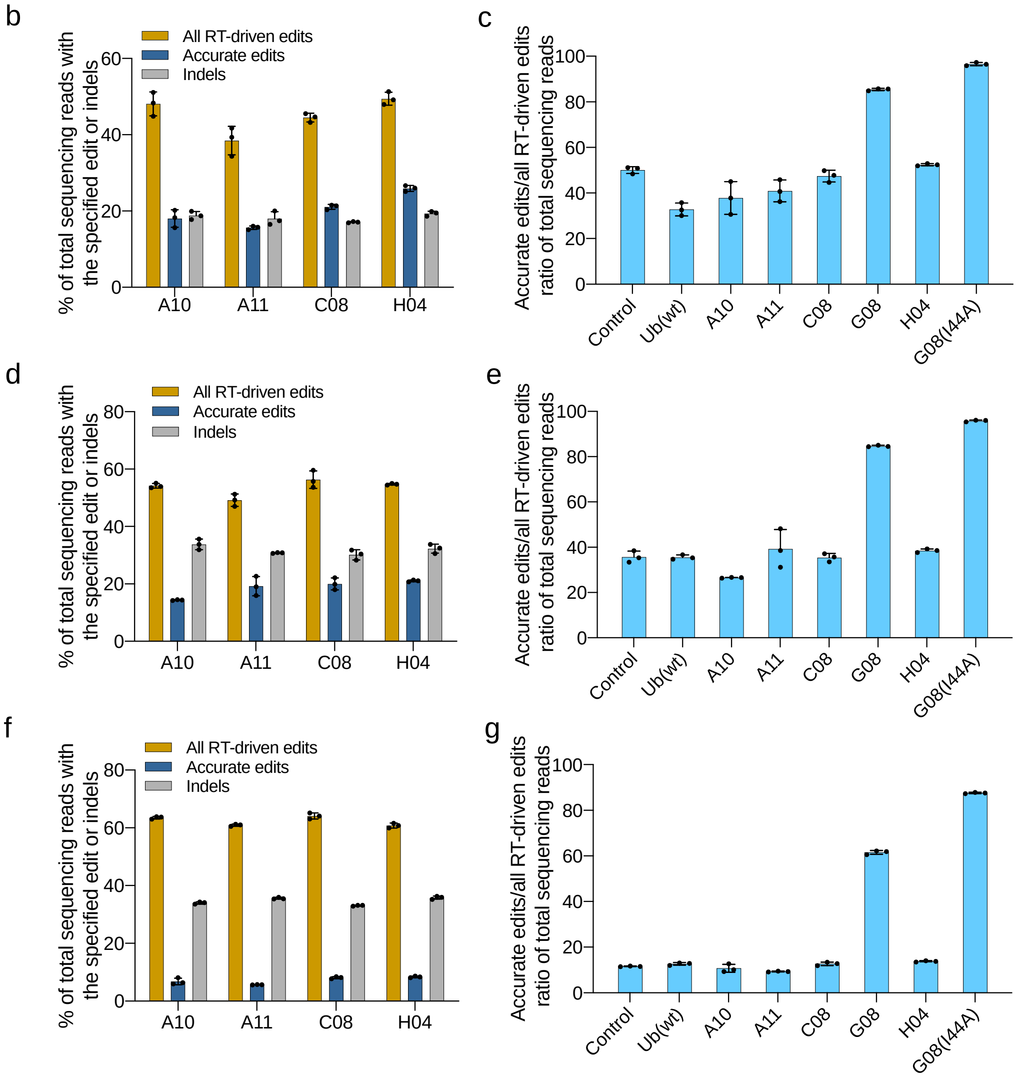

Supplementary Figure 2. Selected 53BP1-targeting ubiquitin variants enhanced PEn-mediated genome editing.

a. Amino acid sequences of the ubiquitin variants.

b. The frequency of all correct edits, accurate edits and indels treated with PE-nuclease following the co-transfection of A10, A11, C08 and H04 ubiquitin variants at

LSP1 site in HEK293T cells. Values and error bars reflect the mean and s.d. of three biological replicates.

c. The ratio of accurate edits relative to all RT-driven edits under all different ubiquitin variants treatment in (**b**) and in Fig. 1b. Values and error bars reflect the mean and s.d. of three biological replicates.

d. The frequency of all correct edits, accurate edits and indels treated with PE-nuclease following the co-transfection of A10, A11, C08 and H04 ubiquitin variants at *SEC61B* site in HEK293T cells. Values and error bars reflect the mean and s.d. of three biological replicates.

e. The ratio of accurate edits relative to all RT-driven edits under different ubiquitin variants treatment in (**d**) and in Fig. 1b. Values and error bars reflect the mean and s.d. of three biological replicates.

f. The frequency of all correct edits, accurate edits and indels treated with PE-nuclease following the co-transfection of A10, A11, C08 and H04 ubiquitin variants at *RUNX1* site in HEK293T cells. Values and error bars reflect the mean and s.d. of three biological replicates.

g. The ratio of accurate edits relative to all RT-driven edits under different ubiquitin variants treatment in (**f**) and in Fig. 1b. Values and error bars reflect the mean and s.d. of three biological replicates.

Source data are provided as a Source Data file.

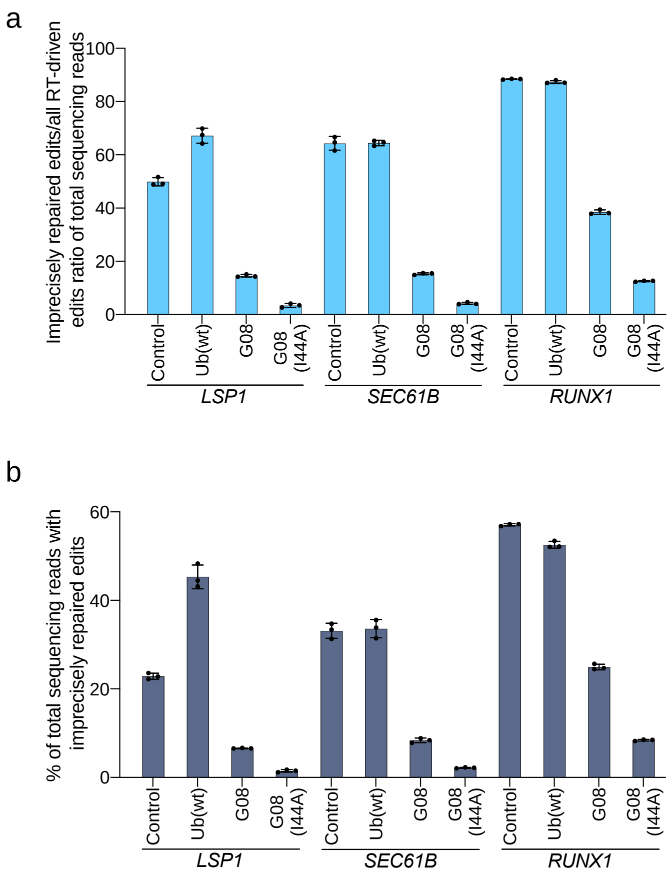

Supplementary Figure 3. 53BP1-targeting ubiquitin variants decreased PEn-associated imprecise edits to improve the purities within RT-driven edits.

a. The ratios of imprecisely repaired edits to all RT-driven edits at *LSP1*, *SEC61B* and *RUNX1* sites in HEK293T cells co-introduced with PEn and the WT Ub, G08 or G08 (I44A) ubiquitin variants. Values and error bars reflect the mean and s.d. of three biological replicates.

b. The frequencies (among all reads) of imprecisely repaired edits at *LSP1*, *SEC61B* and *RUNX1* sites in HEK293T cells co-introduced with PEn and the WT Ub, G08 or G08 (I44A) ubiquitin variants. Values and error bars reflect the means and s.d. of three biological replicates.

Source data are provided as a Source Data file.

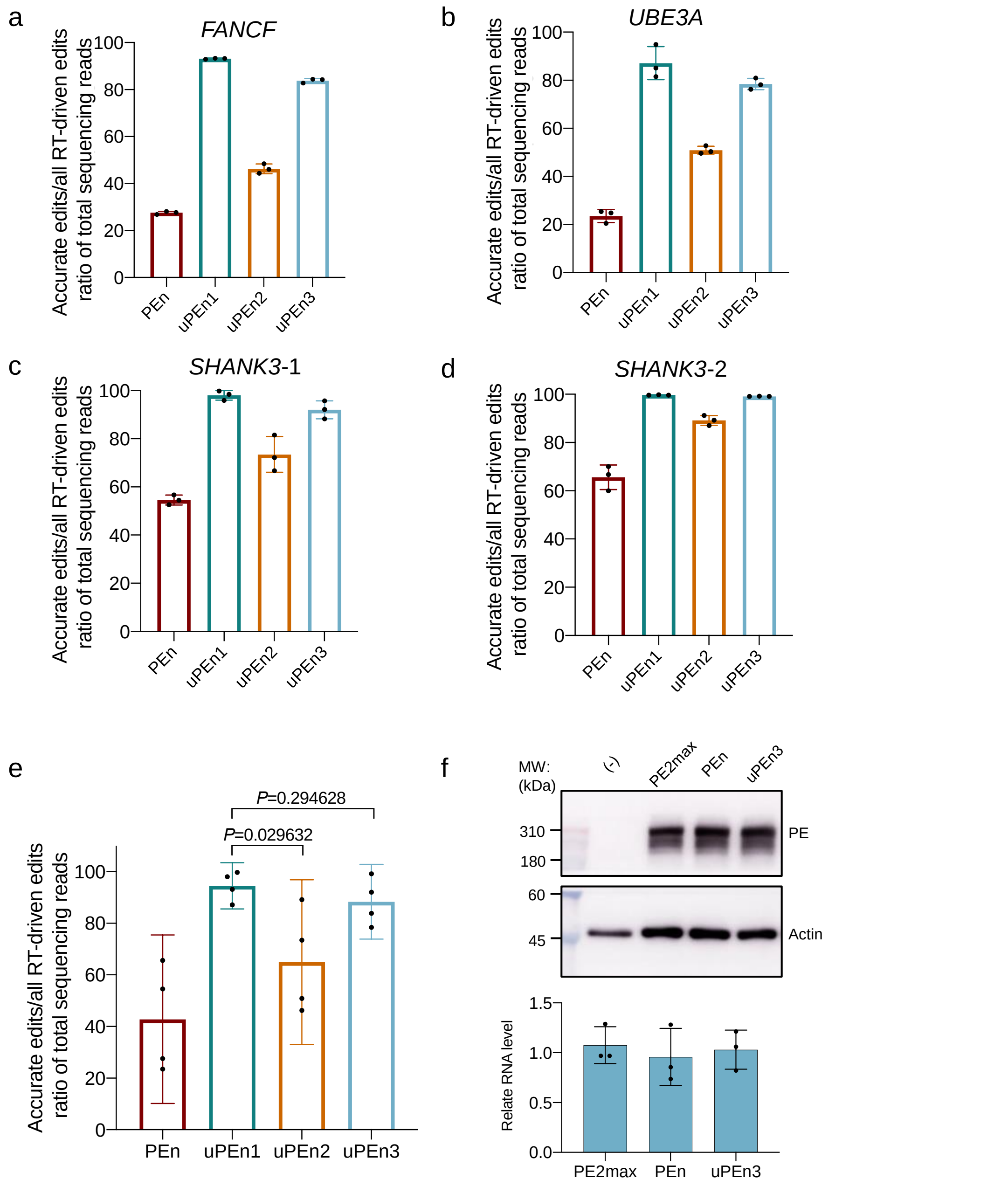

Supplementary Figure 4. The purity of prime editing induced by uPEn system.

a. The ratio of accurate edits relative to all RT-driven edits under the treatment of PEn, uPEn1, uPEn2 and uPEn3 at *FANCF* site. Values and error bars reflect the mean and s.d. of three biological replicates.

b. The ratio of accurate edits relative to all RT-driven edits under the treatment of PEn, uPEn1, uPEn2 and uPEn3 at *UBE3A* site. Values and error bars reflect the mean and s.d. of three biological replicates.

c. The ratio of accurate edits relative to all RT-driven edits under the treatment of PEn, uPEn1, uPEn2 and uPEn3 at *SHANK3*-1 site. Values and error bars reflect the mean and s.d. of three biological replicates.

d. The ratio of accurate edits relative to all RT-driven edits under the treatment of PEn, uPEn1, uPEn2 and uPEn3 at *SHANK3*-2 site. Values and error bars reflect the mean and s.d. of three biological replicates.

e. Statistical analysis of the ratio of accurate edits relative to all RT-driven edits under the treatment of PEn, uPEn1, uPEn2 and uPEn3. The averaged values at 4 different sites from (**a-d**) were summarized. Values and error bars reflect the mean and s.d. of averaged values from editing applications at four different sites. *P* values (directed marked on the graph) were calculated by two-tailed Student's t-tests.

f. HEK293T cells were transfected with PEn, uPEn3 and PE2max (as a control) together with the pegRNA targeting at the *UBE3A* site. The protein samples were analyzed by WB (top). The levels of pegRNA were examined by qPCR (bottom). Values and error bars reflect the mean and s.d. of three biological replicates.

Source data are provided as a Source Data file.

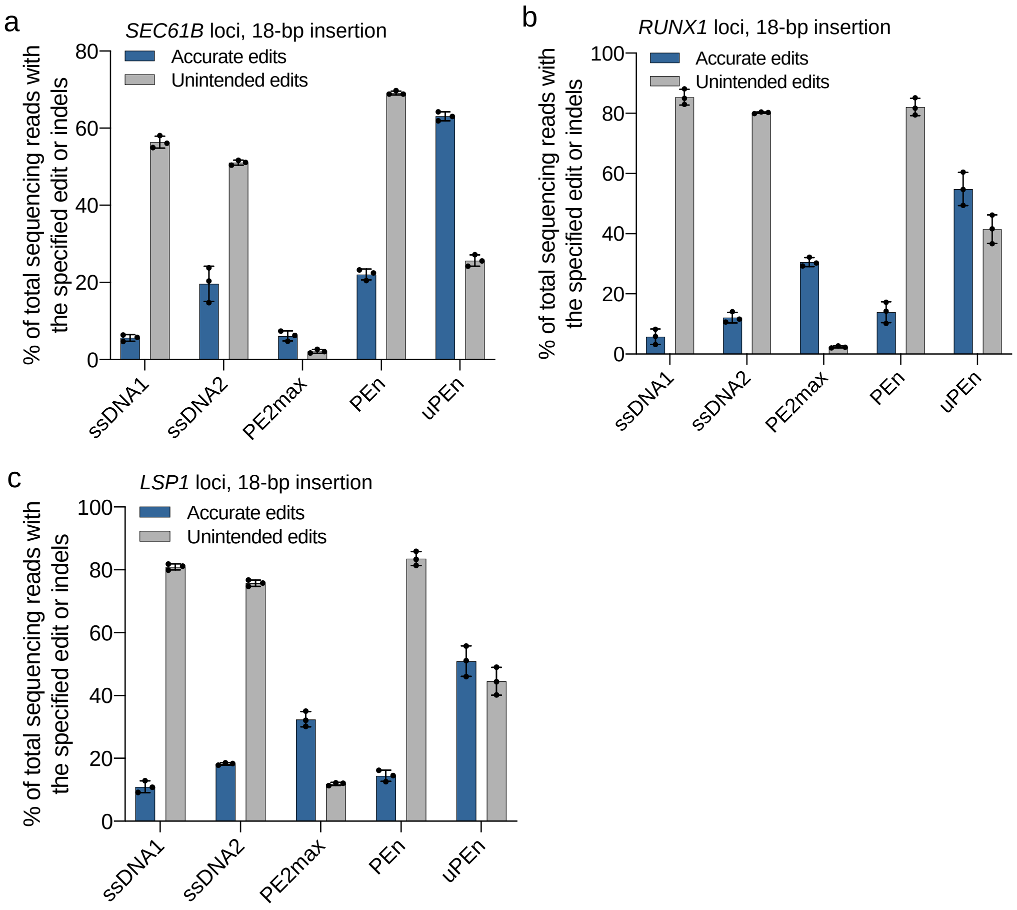

Supplementary Figure 5. The editing efficiencies of PEn/uPEn compared to the Cas9/template and PE2max system.

a. Comparison of 18-bp insertion efficiencies induced by Cas9/ssDNA, PE2max, PEn and uPEn at the *SEC61B* site in HEK293T cells. Values and error bars reflect the means and standard deviation (s.d.) of three biological replicates.

b. Comparison of 18-bp insertion efficiencies induced by Cas9/ssDNA, PE2max, PEn and uPEn at the *RUNX1* site in HEK293T cells. Values and error bars reflect the means and standard deviation (s.d.) of three biological replicates.

c. Comparison of 18-bp insertion efficiencies induced by Cas9/ssDNA, PE2max, PEn and uPEn at the *LSP1* site in HEK293T cells. Values and error bars reflect the means and standard deviation (s.d.) of three biological replicates.

Source data are provided as a Source Data file.

a

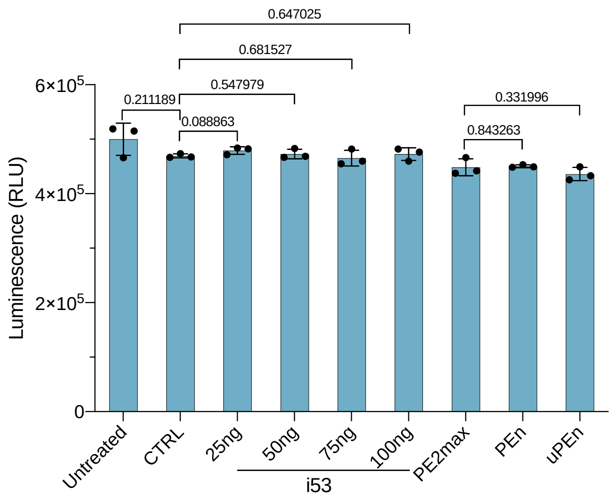

b

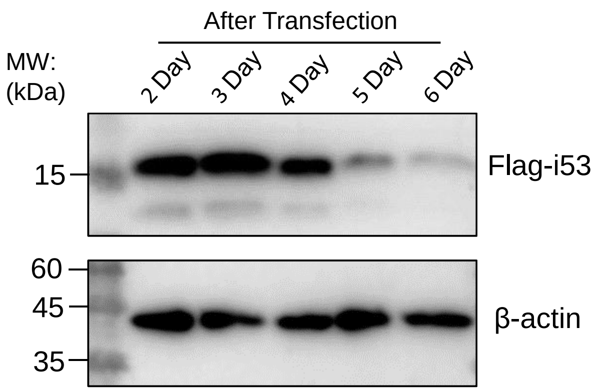

Supplementary Figure 6. Examination of cell viability in response to the transfected i53 or uPEn.

a. Measurements on the viability of HEK293T cells transfected with PE2max, PEn, uPEn or different amounts of i53 in 96-well plates. The assays were carried out using the luminescence detection kit. Values and error bars reflect the means and standard deviation (s.d.) of three biological replicates. *P* values (directed marked on the graph) were calculated by two-tailed Student's t-tests.

b. Western blot analysis of Flag-i53 expression in HEK293T cells at indicated time points after transfection. This control experiment was carried out for one time. Source data are provided as a Source Data file.

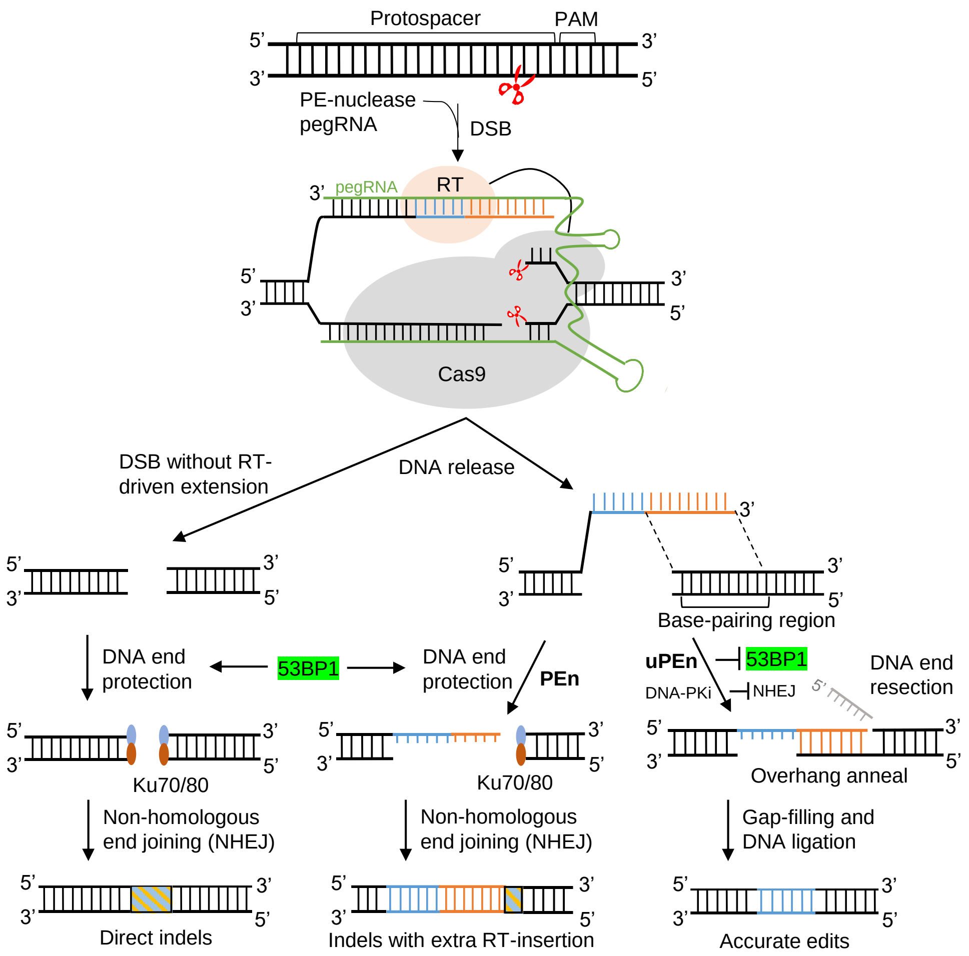

Supplementary Figure 7. The potential mechanism(s) underlying PEn- and uPEn-dependent editing.

An illustration depicting the potential intermediates and repair mechanisms associated with PEn- and uPEn-dependent DSB is shown. The nuclease activity in PEn initially generates a blunt-ended DSB, with the upstream (PAM-distal) end readily undergo pegRNA-programmed reverse transcription to form a 3′ overhang structure (upper part). Note that the blue and orange segments within the overhang structure depict the inserted sequence and the ensuing arm homologous to the downstream DSB end, respectively. If the DSB is repaired prior to the RT action, the NHEJ pathway would lead to classical indel formation (lower left). The Ku70/80 complex formed on the largely unprocessed DSB are marked by oval shapes in blue/brown. Moreover, the indel in the repaired product is indicated by a box with crossbar pattern. On the other hand, with the RT-generated overhang structure, the predominant NHEJ process would attempt to join such RT-driven overhang structure

to the downstream, end-protected DSB terminus (PAM-proximal), causing imprecise edits by PEn (apparent insertion of RTT-derived sequences [in orange] with different lengths). Here, the box with crossbar pattern indicates the imprecise nature of the RTT insertion. Contrastingly, the i53 activity in uPEn (or the use of NHEJ inhibitor) would stimulate the resection of the downstream end of DSB, exposing another 3′ overhang structure capable of specific base-pairing with the homologous (orange) portion of the upstream overhang. This would yield the key intermediate for the eventual installation of precise edits.

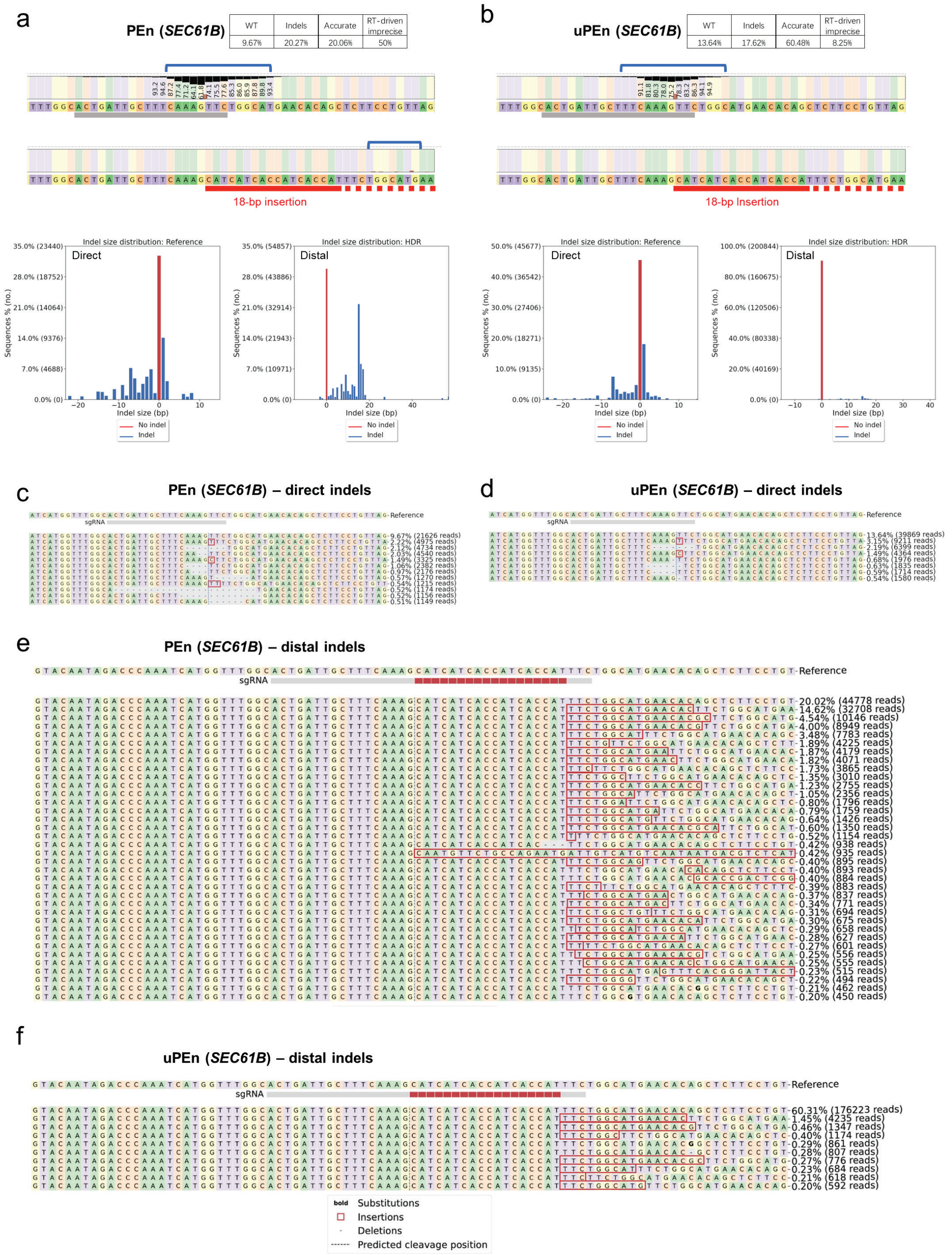

Supplementary Figure 8. CRISPResso2-based analyses of editing outcomes by PEn/uPEn at *SEC61B*.

a and b. The editing outcomes by PEn **(a)** and uPEn **(b)** are presented firstly by the overlay of edits along the reference and the desirable product sequences (top graphs). The guide RNA target site is marked by a thick grey line underneath the reference sequence, while the 18-bp insertion is marked by a thick red line

underneath the desirable product sequence. The dotted red lines indicate the segment of homology. The positions of the indels are highlighted by blue brackets. The “indel size distribution graphs” are shown in the lower portion of the panel. The results for direct and distal indels are presented separately.

c-f. The allele frequencies resulting from PEn **(c, e)** and uPEn **(d, f)** are presented next in the “sequence alignment viewer”. The alleles aligning to the reference sequence are shown in (c) and (d), while those aligning to the desirable product sequence are shown in (e) and (f). In these graphs, the predicted cleavage sites are marked by dotted vertical lines. Red boxes indicate undesirable insertions, while “-“s denote deletions. The substituted nucleotides are shown in bold letters.

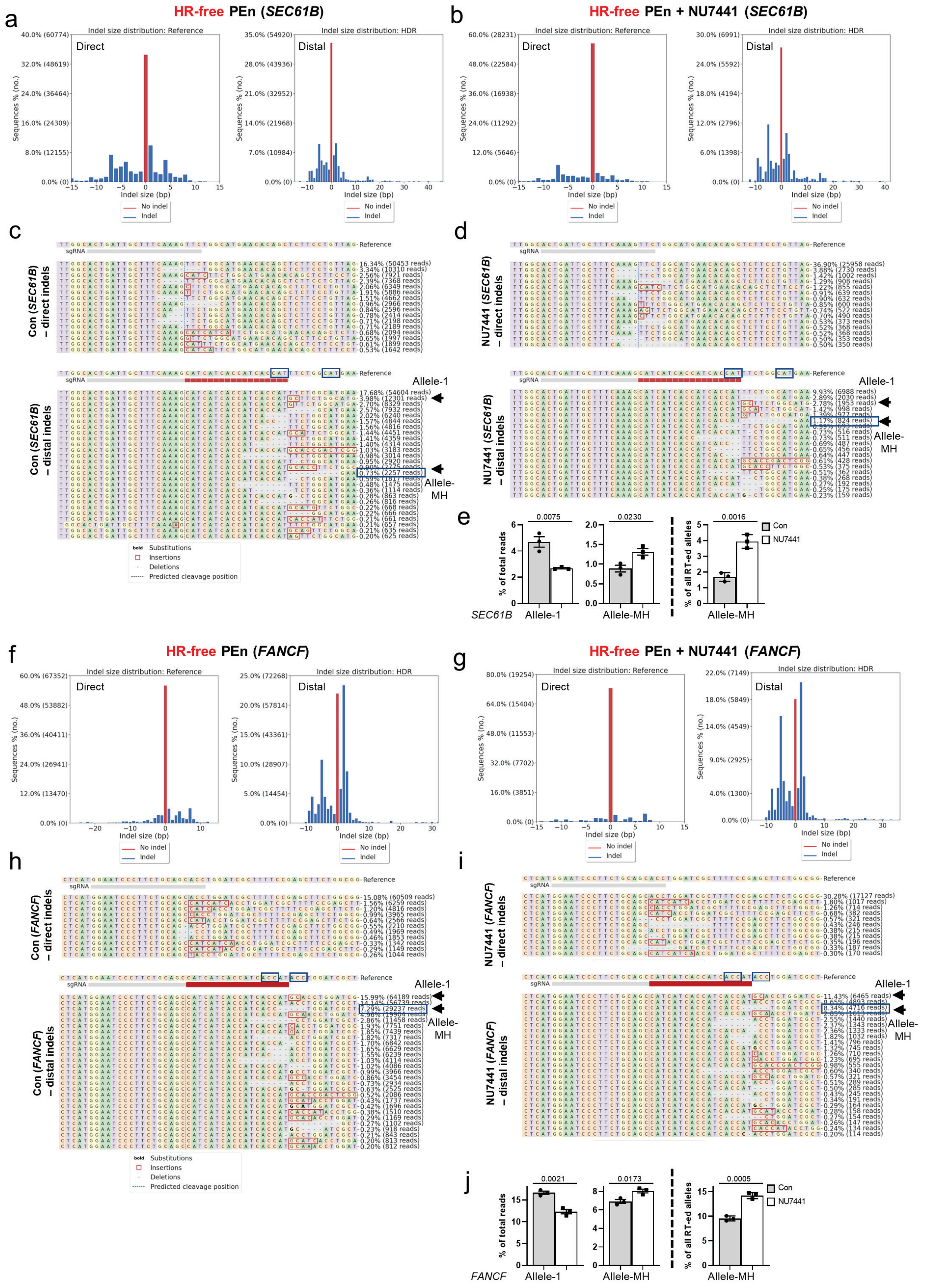

Supplementary Figure 9. CRISPResso2-based analyses of editing outcomes by PEn (± NU7441) with HR-free pegRNAs.

a-e. The editing outcomes at the *SEC61B* site are presented. The “indel size distribution graphs” from the HR-free-type PEn experiments under the control and NU7441-treated conditions are shown respectively in panels (**a**) and (**b**). The

corresponding outputs in the “sequence alignment viewers” are shown in panels (**c**) and (**d**). The microhomologies between the RT-dependent overhang and the downstream DSB end are highlighted by blue boxes. The microhomology-shaped allele (allele-MH) and an unrelated abundant allele (allele-1) are respectively marked by arrowheads. In these graphs, the predicted cleavage sites are marked by dotted vertical lines. Red boxes indicate undesirable insertions, while “-“s denote deletions. The substituted nucleotides are shown in bold letters. In (**e**), the levels of allele-1, allele-MH and the relative levels of the latter to all RT-dependent edits are shown as indicated. Values and error bars reflect the mean and s.d. of three biological replicates. *P* values were calculated by a two-tailed Student’s t-test (marked on the graphs).

f-j. The editing outcomes at the *FANCF* site are presented. The “indel size distribution graphs” from the HR-free-type PEn experiments under the control and NU7441-treated conditions are shown respectively in panels (**f**) and (**g**). The corresponding outputs in the “sequence alignment viewers” are shown in panels (**h**) and (**i**). In (**j**), the levels of allele-1, allele-MH and the relative levels of the latter to all RT-dependent edits are shown as indicated. Values and error bars reflect the mean and s.d. of three biological replicates. *P* values were calculated by a two-tailed Student’s t-test (marked on the graphs).

Source data in (e) and (j) are provided as a Source Data file.

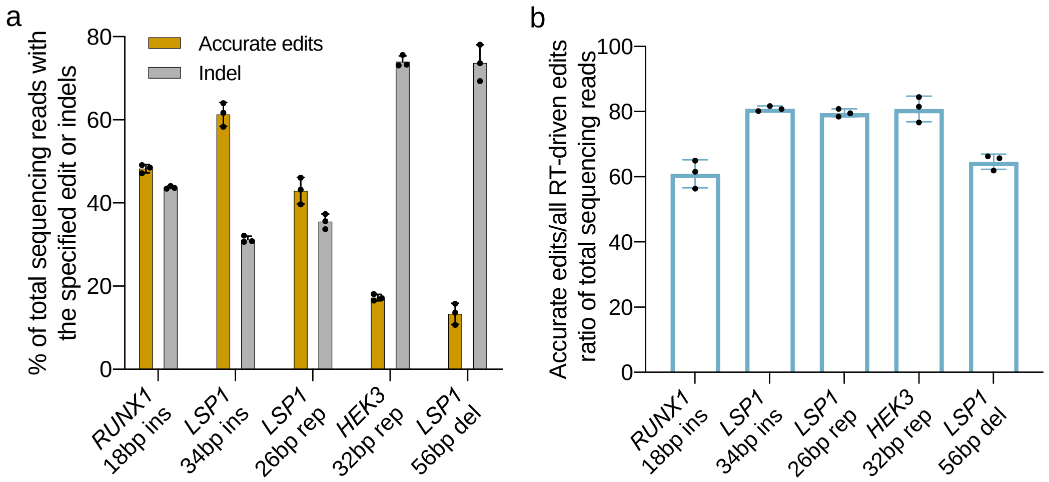

Supplementary Figure 10. Targeted sequence insertion, deletion and replacement by uPEn system in U2OS cells.

a. Targeted insertion, deletion and replacement edits with uPEn at multiple sites in U2OS cells. Values and error bars reflect the mean and s.d. of three biological replicates.

b. The ratio of uPEn-induced accurate edits relative to all RT-driven edits at the targeted sites in **(a)**. Similarly, values and error bars reflect the mean and s.d. of three biological replicates.

Source data are provided as a Source Data file.

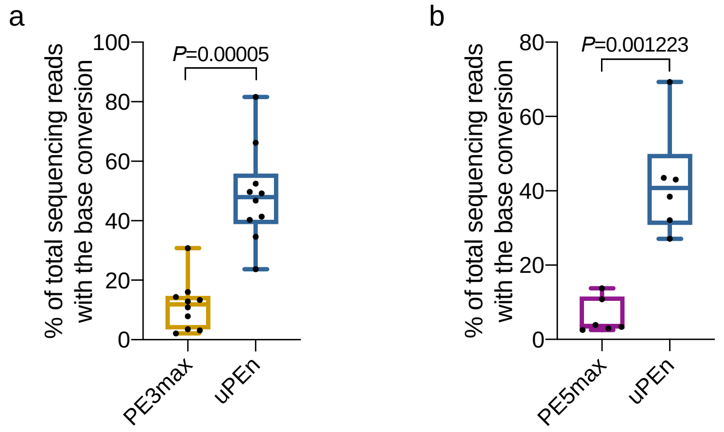

Supplementary Figure 11. Statistical analysis of prime editing efficiency and indels in HEK293 and U2OS cells.

a. Statistical analysis of prime editing efficiency of PE3max and uPEn system in HEK293T cells. The center line shows medians of all data points and the box limits correspond to the upper the lower quartiles, while the whiskers extend to the largest and smallest values. n = 10 (sites) for each group. *P* value (marked directly on the graph) was calculated by a two-tailed Student's t-test.

b. Statistical analysis of desirable base conversion efficiencies of PE5max and uPEn system in U2OS cells. The box plot was generated with the same settings as in (**a**). n = 6 (sites) for each group. *P* value (marked directly on the graph) was calculated by a two-tailed Student's t-test.

Source data are provided as a Source Data file.

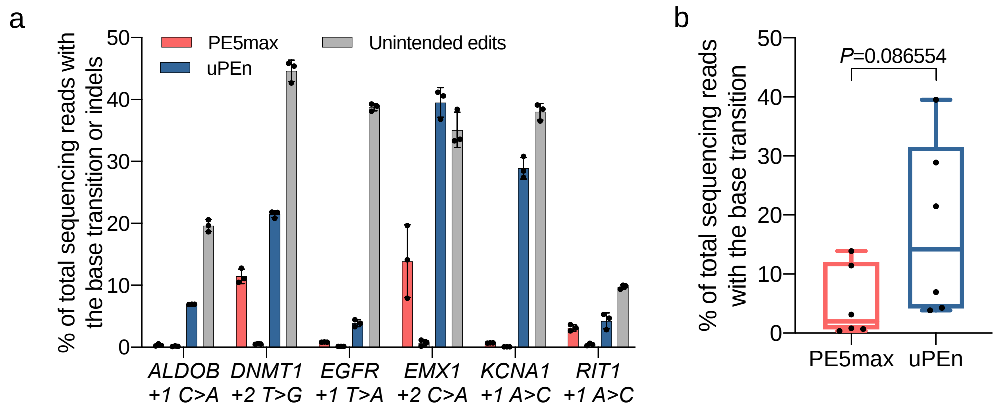

Supplementary Figure 12. Comparisons of uPEn and PE5max for installing base conversions in HeLa cells.

a. Comparison of base conversion efficiencies and the levels of unintended edits by PE5max and uPEn at six endogenous sites in HeLa cells. Values and error bars reflect the mean and s.d. of three biological replicates.

b. Statistical analysis of desirable base conversion efficiencies of PE5max and uPEn in HeLa cells. The center line shows medians of all data points and the box limits correspond to the upper the lower quartiles, while the whiskers extend to the largest and smallest values. n = 6 (sites) for each group. *P* value (marked directly on the graph) was calculated by a two-tailed Student's t-test.

Source data are provided as a Source Data file.

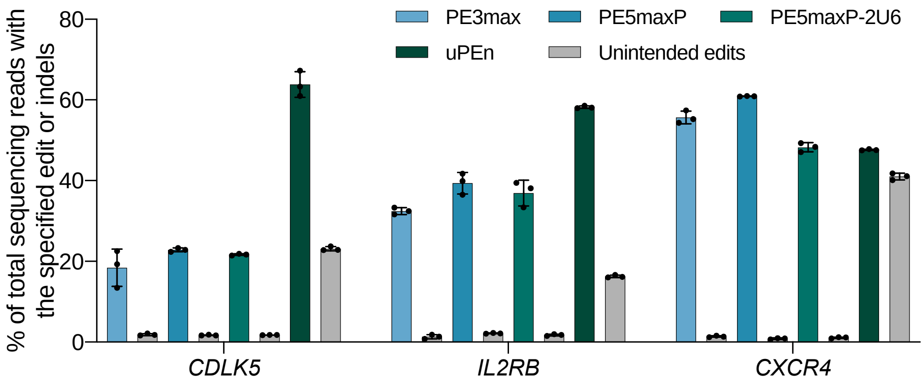

Supplementary Figure 13. Comparisons among uPEn, PE3max and configuration-simplified PE5max for base conversions at previously tested sites.

Comparison of base conversion efficiencies and indels induced by PE3max, PE5maxP, PE5maxP-2U6 and uPEn at three reported sites in HEK293T cells. PE5maxP: a construct with PEmax and the MLH1dn modules connected via P2A. PE5maxP-2U6: PE5maxP transfected together with a plasmid containing both U6-driven pegRNA and nick-sgRNA cassettes. Values and error bars reflect the mean and s.d. of three biological replicates.

Source data are provided as a Source Data file.

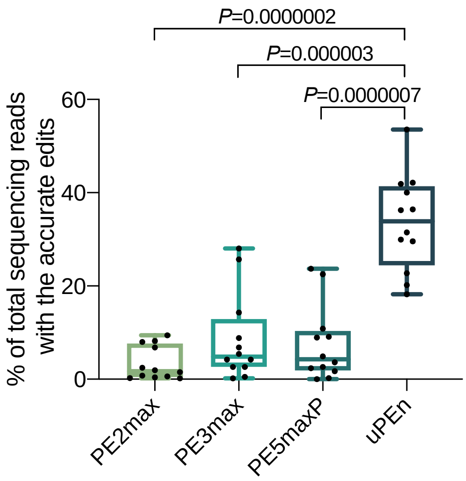

Supplementary Figure 14. Benchmarking the performances of uPEn for insertion, deletion and replacement.

Statistical analyses on the efficiencies of targeted insertion, deletion and replacement by PE2max, PE3max, PE5max and uPEn system in HEK293T cells. The center line shows medians of all data points and the box limits correspond to the upper the lower quartiles, while the whiskers extend to the largest and smallest values. n = 12 (sites) for each group. *P* values (marked directly on the graph) were calculated by two-tailed Student's t-tests. Source data are provided as a Source Data file.

a

FANCF	sgRNA sequence	Chr.	Start position	End position
On	GGAATCCCTTCTGCAGCACC	chr11	22625789	22625808
OT1	GGAACCCCGTCTGCAGCACC	chr18	8707527	8707546
OT2	AGAGGCCCCTCTGCAGCACC	chr17	80950161	80950180
OT3	ACCATCCCTCCTGCAGCACC	chrX	87100160	87100179
OT4	TGAATCCCATCTCCAGCACC	chr10	71703362	71703381

HEK3	sgRNA sequence	Chr.	Start position	End position
On	GGCCCAGACTGAGCACGTGA	chr9	107422339	107422358
OT1	CACCCAGACTGAGCACGTGC	chr15	79457572	79457591
OT2	AGCTCAGACTGAGCAAGTGA	chr1	46540030	46540049
OT3	AGACCAGACTGAGCAAGAGA	chrX	115529809	115529828
OT4	GAGCCAGAATGAGCACGTGA	chr10	129794840	129794859

HEK4	sgRNA sequence	Chr.	Start position	End position
On	GGCACTGCGGCTGGAGGTGG	chr20	32761950	32761969
OT1	TGCACTGCGGCCGGAGGAGG	chr20	61435490	61435509
OT2	GGCTCTGCGGCTGGAGGGGG	chr19	32891172	32891191
OT3	GGCATCACGGCTGGAGGTGG	chr10	75343345	75343364
OT4	GGCGCTGCGGCGGGAGGTGG	chr15	40752041	40752060

b

	FANCF		*HEK3*		*HEK4*	
	PEn	uPEn	PEn	uPEn	PEn	uPEn
On-target	9.78	24.04	3.58	12.12	5.56	19.3
Off-target 1	2.03	3.87	1.50	1.29	34.52	20.42
Off-target 2	0.01	0.03	3.47	5.77	29.01	31.01
Off-target 3	0.02	0.02	0.03	0.14	6.85	4.20
Off-target 4	0.01	0.01	0.31	0.04	44.63	52.20

Color scale: 10, 20, 30, 40, 50

Supplementary Figure 15. Comparison of potential off-target rates for PEn and uPEn at three genomic sites in HEK293T cells.

a. Information for the off-target sites with *FANCF*, *HEK3* and *HEK4* as the desired targets.

b. Off-target analyses for PEn and uPEn-mediated targeting of *FANCF*, *HEK3* and *HEK4*. Values reflect the mean of three biological replicates.

a

Sample	Sequenced reads	Mapping rate	Duplication	Mean depth
Untransfected	604,744,838	95.71%	17.26%	20.34X
Control	564,646,676	98.98%	14.82%	21.67X
PE5max	569,574,624	98.60%	14.83%	20.72X
PEn	577,800,824	98.61%	16.11%	21.38X
uPEn	561,389,458	98.72%	17.71%	21.09X

b

	Control	PE5max	PEn	uPEn
C:G > T:A	19,123	18,453	17,998	17,209
C:G > G:C	4,712	4,453	4,384	4,202
C:G > A:T	5,691	5,553	5,420	5,242
A:T > G:C	16,538	15,901	15,450	14,749
A:T > C:G	4,656	4,738	4,591	4,461
A:T > T:A	4,275	4,142	3,958	3,824
Indels	63,021	61,456	61,519	63,158

c

Mismatch	Predicted	Control	PE5max	PEn	uPEn
1	0	0	0	0	0
2	1	0	0	1	1
3	38	0	0	0	0
4	438	0	0	0	0
5	2,954	0	0	0	0

Supplementary Figure 16. Whole genome sequencing analyses of off-target effects associated with PE5max, PEn and uPEn.

a. Whole-genome sequencing sample information. HEK293T cells were respectively transfected with EGFP (control); PE5max, PEn and uPEn. The *FANCF* site was targeted in cells transfected with various PE platforms.

b. Summary of total unique variants and indels detected by WGS in control, PE5max, PEn, and uPEn samples.

c. Summary of off-target analysis. Potential off-target sites with up to 5-nt mismatches from the spacer of the targeting pegRNA were predicted by Cas-OFFinder (requiring NRG as PAM).

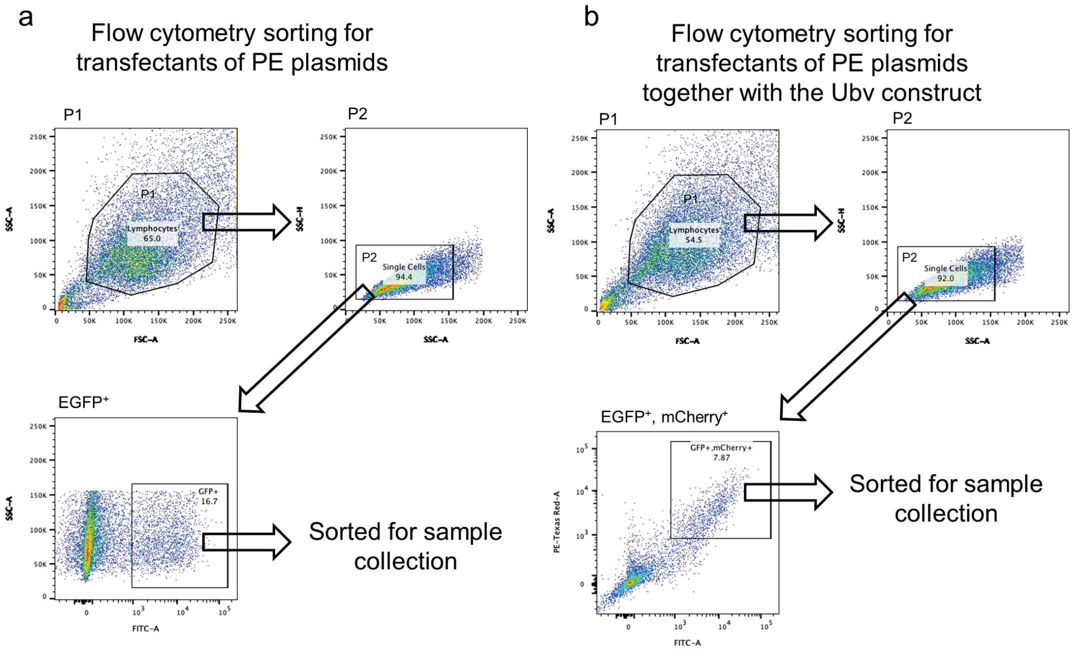

Supplementary Figure 17. Flow cytometry gating strategies.

a. Example of gating strategy for flow cytometry sorting of cells transfected with PE plasmids (PEn, uPEn3, PE2max, PE3max, PE5max, PE5maxP). The transfected cells ells were sorted via sequential gates of live cells (P1), single cells (P2) and positivity of EGFP fluorescence (EGFP$^+$, encoded by the plasmid for pegRNA). This gating strategy corresponds to all editing experiments, except for some samples in Fig. 1 and Supplementary Fig. 2-4 (the transfectants with EGFP$^+$/mCherry$^+$ populations).

b. Example of gating strategy for flow cytometry sorting of cells transfected with PE plasmids together with the Ubv constructs. The P1 gate selects for live cells, the P2 gate selects for single cells. The EGFP$^+$/mCherry$^+$ gate selects for cells that were transfected with PE plasmid and Ubv plasmid. This gating strategy corresponds to results in Fig. 1 and Supplementary Fig. 2-4 (except for some transfectants with EGFP$^+$-only populations).

Supplementary Tables

Supplementary Table 1. Primers used for constructing of pegRNA and HR-pegRNA plasmids.

Backbone pegRNA-F	AGCTAGGTCTCCTTTTTTTAAAGAATTCTCGACCTCGAGAC
Backbone pegRNA-R	TCTCTCGGTCTCACGGTGTTTCGT
sgRNA scaffold-top	AGAGCTAGAAATAGCAAGTTAAAATAAGGCTAGTCCGTTATCAACTTGAAAAAGTGGCACCGAGTCG
sgRNA scaffold-bottom	GCACCGACTCGGTGCCACTTTTTCAAGTTGATAACGGACTAGCCTTATTTTAACTTGCTATTTCTAG

Supplementary Table 2. Primers used for ssDNA donors

LSP1-ssDNA1	GAGAAGGCAACACCATCATCACCATCACCATTGCCGGCTGGGGCTG
LSP1-ssDNA2	GGTTACAAAGGGCTGCCACTGTGAGAAGGCAACACCATCATCACCATCACCATTGCCGGCTGGGGCTGGGCTTTCTACCTCACCAAGC
SEC61B-ssDNA1	GATTGCTTTCAAAGCATCATCACCATCACCATTTCTGGCATGAACA
SEC61B-ssDNA2	CCCAAATCATGGTTTGGCACTGATTGCTTTCAAAGCATCATCACCATCACCATTTCTGGCATGAACACAGCTCTTCCTGTTAGAACCA
RUNX1-ssDNA1	ATTTTCAGGAGGAAGCATCATCACCATCACCATCGATGGCTTCAGA
RUNX1-ssDNA2	CCGCCTTCAGAAGAGGGTGCATTTTCAGGAGGAAGCATCATCACCATCACCATCGATGGCTTCAGACAGCATATTTGAGTCATTTCC

Supplementary Table 3. Primers used for pegRNA RT-qPCR analysis.

NO.	Forward Primer	Reverse Primer
qPCR-UBE3A	CAGGGAAATACTGGAAACTC	ACGATGACAAGCTCGAGGAT
qPCR-Cas9	CTCTGTGGGCTGGGCC	TGCTGTGCCGGTCGG

Supplementary Notes

Supplementary Note 1. The sequence of Blank Control (mCherry).

mCherry

cgtacggccaccatggtgagcaagggcgaggaggataacatggccatcatcaaggagttcatgcgcttc
aaggtgcacatggagggctccgtgaacggccacgagttcgagatcgagggcgagggcgagggccgcc
cctacgagggcacccagaccgccaagctgaaggtgaccaagggtggccccctgcccttcgcctgggac
atcctgtcccctcagttcatgtacggctccaaggcctacgtgaagcaccccgccgacatccccgactacttg
aagctgtccttccccgagggcttcaagtgggagcgcgtgatgaacttcgaggacggcggcgtggtgaccgt
gacccaggactcctccctgcaggacggcgagttcatctacaaggtgaagctgcgcggcaccaacttcccc
tccgacggccccgtaatgcagaagaagaccatgggctgggaggcctcctccgagcggatgtaccccga
ggacggcgccctgaagggcgagatcaagcagaggctgaagctgaaggacggcggccactacgacgct
gaggtcaagaccacctacaaggccaagaagcccgtgcagctgcccggcgcctacaacgtcaacatcaa
gttggacatcacctcccacaacgaggactacaccatcgtggaacagtacgaacgcgccgagggccgcc
actccaccggcggcatggacgagctgtacaagtaa

Supplementary Note 2. The sequence of mCherry-P2A-Ub (WT).

mCherry
P2A
NLS
Ubiquitin

cgtacggccaccatggtgagcaagggcgaggaggataacatggccatcatcaaggagttcatgcgcttc
aaggtgcacatggagggctccgtgaacggccacgagttcgagatcgagggcgagggcgagggccgcc
cctacgagggcacccagaccgccaagctgaaggtgaccaagggtggccccctgcccttcgcctgggac
atcctgtcccctcagttcatgtacggctccaaggcctacgtgaagcaccccgccgacatccccgactacttg
aagctgtccttccccgagggcttcaagtgggagcgcgtgatgaacttcgaggacggcggcgtggtgaccgt
gacccaggactcctccctgcaggacggcgagttcatctacaaggtgaagctgcgcggcaccaacttcccc
tccgacggccccgtaatgcagaagaagaccatgggctgggaggcctcctccgagcggatgtaccccga
ggacggcgccctgaagggcgagatcaagcagaggctgaagctgaaggacggcggccactacgacgct
gaggtcaagaccacctacaaggccaagaagcccgtgcagctgcccggcgcctacaacgtcaacatcaa
gttggacatcacctcccacaacgaggactacaccatcgtggaacagtacgaacgcgccgagggccgcc
actccaccggcggcatggacgagctgtacaagggatccggcgcaacaaacttctctctgctgaaacaag
ccggagatgtcgaagagaatcctggaccgatgcatatgaaacggacagccgacggaagcgagttcgag
tcaccaaagaagaagcggaaagtcgccgctagcctgaatggagcccctctgatcaaggaccccatgca
gatcttcgtgaagaccctgaccggcaagacaatcaccctggaggtggaaccttctgacaccatcgagaac
gtgaaggccaagatccaggacaaggagggcatcccacctgatcagcagagactgattttcgccggcaaa
cagctggaagatggcagaaccctctccgactacaacatccaaaaagagagcacactgcacctggtcctg
cggctgagataa

Supplementary Note 3. The sequence of mCherry-P2A-H04.

mCherry
P2A
NLS

Ubiquitin
cgtacggccaccatggtgagcaagggcgaggaggataacatggccatcatcaaggagttcatgcgcttc
aaggtgcacatggagggctccgtgaacggccacgagttcgagatcgagggcgagggcgagggccgcc
cctacgagggcacccagaccgccaagctgaaggtgaccaagggtggccccctgcccttcgcctgggac
atcctgtcccctcagttcatgtacggctccaaggcctacgtgaagcaccccgccgacatccccgactacttg
aagctgtccttccccgagggcttcaagtgggagcgcgtgatgaacttcgaggacggcggcgtggtgaccgt
gacccaggactcctccctgcaggacggcgagttcatctacaaggtgaagctgcgcggcaccaacttcccc
tccgacggccccgtaatgcagaagaagaccatgggctgggaggcctcctccgagcggatgtaccccga
ggacggcgccctgaagggcgagatcaagcagaggctgaagctgaaggacggcggccactacgacgct
gaggtcaagaccacctacaaggccaagaagcccgtgcagctgcccggcgcctacaacgtcaacatcaa
gttggacatcacctcccacaacgaggactacaccatcgtggaacagtacgaacgcgccgagggccgcc
actccaccggcggcatggacgagctgtacaagggatccggcgcaacaaacttctctctgctgaaacaag
ccggagatgtcgaagagaatcctggaccgatgcatatgaaacggacagccgacggaagcgagttcgag
tcaccaaagaagaagcggaaagtcgccgcttctctgaacggcgcccctctgatcaaggaccctatgcgg
atcatcgtgaaaaccttcatgagaaagcctattacactcgaggtggaaccatccgacaccatcgagaatgt
gaaggccaagatccaagataaggagggcatccctcccgaccagcagagactgtacttcgccgccagcc
agctggaagatggaagaacactgagcgactacaacatccagaaagagagcaccctgctgctggtcgtg
cggctgctgagagtgtaa

Supplementary Note 4. The sequence of mCherry-P2A-A10.

mCherry
P2A
NLS
Ubiquitin
cgtacggccaccatggtgagcaagggcgaggaggataacatggccatcatcaaggagttcatgcgcttc
aaggtgcacatggagggctccgtgaacggccacgagttcgagatcgagggcgagggcgagggccgcc
cctacgagggcacccagaccgccaagctgaaggtgaccaagggtggccccctgcccttcgcctgggac
atcctgtcccctcagttcatgtacggctccaaggcctacgtgaagcaccccgccgacatccccgactacttg
aagctgtccttccccgagggcttcaagtgggagcgcgtgatgaacttcgaggacggcggcgtggtgaccgt
gacccaggactcctccctgcaggacggcgagttcatctacaaggtgaagctgcgcggcaccaacttcccc
tccgacggccccgtaatgcagaagaagaccatgggctgggaggcctcctccgagcggatgtaccccga
ggacggcgccctgaagggcgagatcaagcagaggctgaagctgaaggacggcggccactacgacgct
gaggtcaagaccacctacaaggccaagaagcccgtgcagctgcccggcgcctacaacgtcaacatcaa
gttggacatcacctcccacaacgaggactacaccatcgtggaacagtacgaacgcgccgagggccgcc
actccaccggcggcatggacgagctgtacaagggatccggcgcaacaaacttctctctgctgaaacaag
ccggagatgtcgaagagaatcctggaccgatgcatatgaaacggacagccgacggaagcgagttcgag
tcaccaaagaagaagcggaaagtcgctgcctccctgaacggagcccctctgatcaaggaccccatgcag
atctacgtgaaaacatttgccagaaagcctatcaccctcgaggtggaacccagcgacaccatcgagaac
gtgaaggccaagatccaggacaaggagggcatccctccagaccagcaaagactgattttcgccgagatg
cggctggaagatggcagaacactgtctgattacaacatcaaaaacgacagcaagctgttcctggtcctga
agaatagcgtgacctaa

Supplementary Note 5. The sequence of mCherry-P2A-A11.

mCherry

P2A
NLS
Ubiquitin
cgtacggccaccatggtgagcaagggcgaggaggataacatggccatcatcaaggagttcatgcgcttc
aaggtgcacatggagggctccgtgaacggccacgagttcgagatcgagggcgagggcgagggccgcc
cctacgagggcacccagaccgccaagctgaaggtgaccaagggtggccccctgcccttcgcctgggac
atcctgtcccctcagttcatgtacggctccaaggcctacgtgaagcaccccgccgacatccccgactacttg
aagctgtccttccccgagggcttcaagtgggagcgcgtgatgaacttcgaggacggcggcgtggtgaccgt
gacccaggactcctccctgcaggacggcgagttcatctacaaggtgaagctgcgcggcaccaacttcccc
tccgacggccccgtaatgcagaagaagaccatgggctgggaggcctcctccgagcggatgtaccccga
ggacggcgccctgaagggcgagatcaagcagaggctgaagctgaaggacggcggccactacgacgct
gaggtcaagaccacctacaaggccaagaagcccgtgcagctgcccggcgcctacaacgtcaacatcaa
gttggacatcacctcccacaacgaggactacaccatcgtggaacagtacgaacgcgccgagggccgcc
actccaccggcggcatggacgagctgtacaagggatccggcgcaacaaacttctctctgctgaaacaag
ccggagatgtcgaagagaatcctggaccgatgcatatgaaacggacagccgacggaagcgagttcgag
tcaccaaagaagaagcggaaagtcgccgcttctctgaacggcgcccctctgatcaaggatcctatgctgat
tttcgtgaccaccgacatgggcatgacaatcagcctcgaggtggaacctagcgacaccatcgagaacgtg
aaggccaagatccaggacaaagagggcatccctccagaccagcagcggctgatcttcggcgacaaag
acctggaagatggaagaacactgtccgattacaacatccaaaaggagagcagcctgaatctggtcctga
agctgagataa

Supplementary Note 6. The sequence of mCherry-P2A-C08.

mCherry
P2A
NLS
Ubiquitin
cgtacggccaccatggtgagcaagggcgaggaggataacatggccatcatcaaggagttcatgcgcttc
aaggtgcacatggagggctccgtgaacggccacgagttcgagatcgagggcgagggcgagggccgcc
cctacgagggcacccagaccgccaagctgaaggtgaccaagggtggccccctgcccttcgcctgggac
atcctgtcccctcagttcatgtacggctccaaggcctacgtgaagcaccccgccgacatccccgactacttg
aagctgtccttccccgagggcttcaagtgggagcgcgtgatgaacttcgaggacggcggcgtggtgaccgt
gacccaggactcctccctgcaggacggcgagttcatctacaaggtgaagctgcgcggcaccaacttcccc
tccgacggccccgtaatgcagaagaagaccatgggctgggaggcctcctccgagcggatgtaccccga
ggacggcgccctgaagggcgagatcaagcagaggctgaagctgaaggacggcggccactacgacgct
gaggtcaagaccacctacaaggccaagaagcccgtgcagctgcccggcgcctacaacgtcaacatcaa
gttggacatcacctcccacaacgaggactacaccatcgtggaacagtacgaacgcgccgagggccgcc
actccaccggcggcatggacgagctgtacaagggatccggcgcaacaaacttctctctgctgaaacaag
ccggagatgtcgaagagaatcctggaccgatgcatatgaaacggacagccgacggaagcgagttcgag
tcaccaaagaagaagcggaaagtcgccgcttctctgaacggcgcccctctgatcaaggatcctatgcaga
tcttcgtcaccaccgacatgtggatgcggatcagcctcgaggtggaaccaagcgacaccatcgagaacgt
gaaggccaagatccaagataaggagggcatccctcccgaccagcagagactgattttcggcgacaagg
acctggaagatggcagaacactgtccgactacaacatccagaaagagagcagcctgaacctggtgctga
atctgagataa

Supplementary Note 7. The sequence of mCherry-P2A-G08.

mCherry
P2A
NLS
Ubiquitin

cgtacggccaccatggtgagcaagggcgaggaggataacatggccatcatcaaggagttcatgcgcttc
aaggtgcacatggagggctccgtgaacggccacgagttcgagatcgagggcgagggcgagggccgcc
cctacgagggcacccagaccgccaagctgaaggtgaccaagggtggcccctgcccttcgcctgggac
atcctgtcccctcagttcatgtacggctccaaggcctacgtgaagcaccccgccgacatccccgactacttg
aagctgtccttccccgagggcttcaagtgggagcgcgtgatgaacttcgaggacggcggcgtggtgaccgt
gacccaggactcctccctgcaggacggcgagttcatctacaaggtgaagctgcgcggcaccaacttcccc
tccgacggccccgtaatgcagaagaagaccatgggctgggaggcctcctccgagcggatgtaccccga
ggacggcgccctgaagggcgagatcaagcagaggctgaagctgaaggacggcggccactacgacgct
gaggtcaagaccacctacaaggccaagaagcccgtgcagctgcccggcgcctacaacgtcaacatcaa
gttggacatcacctcccacaacgaggactacaccatcgtggaacagtacgaacgcgccgagggccgcc
actccaccggcggcatggacgagctgtacaagggatccggcgcaacaaacttctctctgctgaaacaag
ccggagatgtcgaagagaatcctggaccgatgcatatgaaacggacagccgacggaagcgagttcgag
tcaccaaagaagaagcggaaagtcgccgcttctctgaacggcgcccctctgatcaaggatcctatgctgat
cttcgtgaaaacactgacaggcaagaccatcaccctcgaggtggaacctagcgacaccatcgagaatgt
gaaggccaagatccaggacaaggagggcatccctcccgaccagcagagactgattttcgccggaaaaa
gcctggaagatggcagaaccctgtccgactacaacatcctgaaggacagcaagctgcacccactgctgc
ggctgagataa

Supplementary Note 8. The sequence of mCherry-P2A-G08(I44A).

mCherry
P2A
NLS
Ubiquitin

cgtacggccaccatggtgagcaagggcgaggaggataacatggccatcatcaaggagttcatgcgcttc
aaggtgcacatggagggctccgtgaacggccacgagttcgagatcgagggcgagggcgagggccgcc
cctacgagggcacccagaccgccaagctgaaggtgaccaagggtggcccctgcccttcgcctgggac
atcctgtcccctcagttcatgtacggctccaaggcctacgtgaagcaccccgccgacatccccgactacttg
aagctgtccttccccgagggcttcaagtgggagcgcgtgatgaacttcgaggacggcggcgtggtgaccgt
gacccaggactcctccctgcaggacggcgagttcatctacaaggtgaagctgcgcggcaccaacttcccc
tccgacggccccgtaatgcagaagaagaccatgggctgggaggcctcctccgagcggatgtaccccga
ggacggcgccctgaagggcgagatcaagcagaggctgaagctgaaggacggcggccactacgacgct
gaggtcaagaccacctacaaggccaagaagcccgtgcagctgcccggcgcctacaacgtcaacatcaa
gttggacatcacctcccacaacgaggactacaccatcgtggaacagtacgaacgcgccgagggccgcc
actccaccggcggcatggacgagctgtacaagggatccggcgcaacaaacttctctctgctgaaacaag
ccggagatgtcgaagagaatcctggaccgatgcatatgaaacggacagccgacggaagcgagttcgag
tcaccaaagaagaagcggaaagtcgccgccagtttaaacggcgcgccattaattaaggatccaatgttga
ttttcgtgaaaacccttaccgggaaaaccatcaccctcgaggttgaaccctcggatacgatagaaaatgtaa
aggccaagatccaggataaggaaggaattcctcctgatcagcagagactggcctttgctggcaaatcgct
ggaagatggacgtactttgtctgactacaatattctaaaggactctaaacttcatcctctgttgagacttcgttaa

Supplementary Note 9. The sequence of PEn.

NLS

Cas9 nuclease

Linker

Reverse transcriptase

Atgaaacggacagccgacggaagcgagttcgagtcaccaaagaagaagcggaaagtcgacaagaa
gtacagcatcggcctggacatcggcaccaactctgtgggctgggccgtgatcaccgacgagtacaaggtg
cccagcaagaaattcaaggtgctgggcaacaccgaccggcacagcatcaagaagaacctgatcggag
ccctgctgttcgacagcggcgaaacagccgaggccacccggctgaagagaaccgccagaagaagata
caccagacggaagaaccggatctgctatctgcaagagatcttcagcaacgagatggccaaggtggacg
acagcttcttccacagactggaagagtccttcctggtggaagaggataagaagcacgagcggcaccccat
cttcggcaacatcgtggacgaggtggcctaccacgagaagtaccccaccatctaccacctgagaaagaa
actggtggacagcaccgacaaggccgacctgcggctgatctatctggccctggcccacatgatcaagttcc
ggggccacttcctgatcgagggcgacctgaaccccgacaacagcgacgtggacaagctgttcatccagct
ggtgcagacctacaaccagctgttcgaggaaaaccccatcaacgccagcggcgtggacgccaaggcca
tcctgtctgccagactgagcaagagcagaaagctggaaaatctgatcgcccagctgcccggcgagaaga
agaatggcctgttcggaaacctgattgccctgagcctgggcctgacccccaacttcaagagcaacttcgac
ctggccgaggatgccaaactgcagctgagcaaggacacctacgacgacgacctggacaacctgctggc
ccagatcggcgaccagtacgccgacctgtttctggccgccaagaacctgtccgacgccatcctgctgagc
gacatcctgagagtgaacaccgagatcaccaaggcccccctgagcgcctctatgatcaagagatacgac
gagcaccaccaggacctgaccctgctgaaagctctcgtgcggcagcagctgcctgagaagtacaaaga
gattttcttcgaccagagcaagaacggctacgccggctacattgacggcggagccagccaggaagagttc
tacaagttcatcaagcccatcctggaaaagatggacggcaccgaggaactgctcgtgaagctgaagaga
gaggacctgctgcggaagcagcggaccttcgacaacggcagcatcccccaccagatccacctgggaga
gctgcacgccattctgcggcggcaggaagatttttacccattcctgaaggacaaccgggaaaagatcgag
aagatcctgaccttccgcatcccctactacgtgggccctctggccaggggaaacagcagattcgcctggat
gaccagaaagagcgaggaaaccatcaccccctggaacttcgaggaagtggtggacaagggcgcttccg
cccagagcttcatcgagcggatgaccaacttcgataagaacctgcccaacgagaaggtgctgcccaagc
acagcctgctgtacgagtacttcaccgtgtataacgagctgaccaaagtgaaatacgtgaccgagggaat
gagaaagcccgccttcctgagcggcgagcagaaaaaggccatcgtggacctgctgttcaagaccaacc
ggaaagtgaccgtgaagcagctgaaagaggactacttcaagaaaatcgagtgcttcgactccgtggaaat
ctccggcgtggaagatcggttcaacgcctccctgggcacataccacgatctgctgaaaattatcaaggaca
aggacttcctggacaatgaggaaaacgaggacattctggaagatatcgtgctgaccctgacactgtttgag
gacagagagatgatcgaggaacggctgaaaacctatgcccacctgttcgacgacaaagtgatgaagca
gctgaagcggcggagatacaccggctggggcaggctgagccggaagctgatcaacggcatccgggac
aagcagtccggcaagacaatcctggatttcctgaagtccgacggcttcgccaacagaaacttcatgcagct
gatccacgacgacagcctgacctttaaagaggacatccagaaagcccaggtgtccggccagggcgata
gcctgcacgagcacattgccaatctggccggcagccccgccattaagaagggcatcctgcagacagtga
aggtggtggacgagctcgtgaaagtgatgggccggcacaagcccgagaacatcgtgatcgaaatggcc
agagagaaccagaccacccagaagggacagaagaacagccgcgagagaatgaagcggatcgaag
agggcatcaaagagctgggcagccagatcctgaaagaacaccccgtggaaaacacccagctgcagaa
cgagaagctgtacctgtactacctgcagaatgggcgggatatgtacgtggaccaggaactggacatcaac
cggctgtccgactacgatgtggacgctatcgtgcctcagagctttctgaaggacgactccatcgacaacaa

ggtgctgaccagaagcgacaagaaccggggcaagagcgacaacgtgccctccgaagaggtcgtgaa
gaagatgaagaactactggcggcagctgctgaacgccaagctgattacccagagaaagttcgacaatct
gaccaaggccgagagaggcggcctgagcgaactggataaggccggcttcatcaagagacagctggtg
gaaacccggcagatcacaaagcacgtggcacagatcctggactcccggatgaacactaagtacgacga
gaatgacaagctgatccgggaagtgaaagtgatcaccctgaagtccaagctggtgtccgatttccggaag
gatttccagttttacaaagtgcgcgagatcaacaactaccaccacgcccacgacgcctacctgaacgccgt
cgtgggaaccgccctgatcaaaaagtaccctaagctggaaagcgagttcgtgtacggcgactacaaggt
gtacgacgtgcggaagatgatcgccaagagcgagcaggaaatcggcaaggctaccgccaagtacttctt
ctacagcaacatcatgaactttttcaagaccgagattaccctggccaacggcgagatccggaagcggcct
ctgatcgagacaaacggcgaaaccggggagatcgtgtgggataagggccgggattttgccaccgtgcgg
aaagtgctgagcatgccccaagtgaatatcgtgaaaaagaccgaggtgcagacaggcggcttcagcaa
agagtctatcctgcccaagaggaacagcgataagctgatcgccagaaagaaggactgggaccctaaga
agtacggcggcttcgacagccccaccgtggcctattctgtgctggtggtggccaaagtggaaaagggcaa
gtccaagaaactgaagagtgtgaaagagctgctggggatcaccatcatggaaagaagcagcttcgaga
agaatcccatcgactttctggaagccaagggctacaaagaagtgaaaaaggacctgatcatcaagctgc
ctaagtactccctgttcgagctggaaaacggccggaagagaatgctggcctctgccggcgaactgcagaa
gggaaacgaactggccctgccctccaaatatgtgaacttcctgtacctggccagccactatgagaagctga
agggctcccccgaggataatgagcagaaacagctgtttgtggaacagcacaagcactacctggacgag
atcatcgagcagatcagcgagttctccaagagagtgatcctggccgacgctaatctggacaaagtgctgtc
cgcctacaacaagcaccgggataagcccatcagagagcaggccgagaatatcatccacctgtttaccctg
accaatctgggagcccctgccgccttcaagtactttgacaccaccatcgaccggaagaggtacaccagca
ccaaagaggtgctggacgccaccctgatccaccagagcatcaccggcctgtacgagacacggatcgac
ctgtctcagctgggaggtgactccggcggaagctctggtggcagcaagcggaccgccgacggctctgaat
tcgagagccctaagaagaaaagaaaggtgagcggaggctctagcggcggaagcaccctgaacattga
agacgagtatagactgcatgaaacaagcaaggaacccgacgtgtccctgggctccacctggctgtccga
ctttccccaggcctgggccgagacaggaggaatgggcctggccgtgcggcaggcacccctgatcatccct
ctgaaggccacctctacacccgtgagcatcaagcagtaccctatgtctcaggaggccagactgggcatca
agcctcacatccagaggctgctggaccagggcatcctggtgccatgccagagcccctggaacacaccac
tgctgcccgtgaagaagccaggcaccaatgactatagacccgtgcaggatctgagagaggtgaacaag
agggtggaggatatccaccccaccgtgcccaacccttacaatctgctgtccggcctgccccccttctcaccag
tggtatacagtgctggacctgaaggatgccttcttttgtctgagactgcaccctaccagccagccactgttcgc
ctttgagtggagggaccctgagatgggcatctctggccagctgacctggacacgcctgcctcagggcttca
agaatagcccaacactgtttaacgaggccctgcaccgcgacctggcagatttccggatccagcacccaga
tctgatcctgctgcagtacgtggacgatctgctgctggccgccaccagcgagctggattgccagcagggaa
cacgcgccctgctgcagaccctgggaaacctgggatatagggcatccgccaagaaggcccagatctgtc
agaagcaggtgaagtacctgggctatctgctgaaggagggccagagatggctgacagaggccaggaa
ggagacagtgatgggccagccaacacccaagaccccaagacagctgagggagttcctgggcaaagca
ggattttgcaggctgttcatcccaggattcgcagagatggcagcacctctgtacccactgaccaagccggg
caccctgtttaattggggccctgaccagcagaaggcctatcaggagatcaagcaggccctgctgacagca
ccagccctgggcctgccagacctgaccaagcctttcgagctgtttgtggatgagaagcagggctacgcca
agggcgtgctgacccagaagctgggaccatggagacggcccgtggcctatctgtccaagaagctggacc
cagtggcagcaggatggccaccatgcctgaggatggtggcagcaatcgccgtgctgacaaaggatgccg
gcaagctgaccatgggacagccactggtcatcctggcaccacacgcagtggaggccctggtgaagcag
cctccagatcgctggctgtctaacgcccggatgacacactaccaggccctgctgctggacaccgatcgcgt

gcagtttggccctgtggtggccctgaatccagccaccctgctgcctctgccagaggagggcctgcagcaca
actgtctggacatcctggcagaggcacacggaacaaggccagacctgaccgatcagcccctgcctgacg
ccgatcacacatggtataccgatggaagctccctgctgcaggagggccagaggaaggcaggagcagca
gtgaccacagagacagaagtgatctgggccaaggccctgccagcaggcacatccgcccagcgggccg
agctgatcgccctgacccaggccctgaagatggccgagggcaagaagctgaacgtgtacacagactcc
agatatgccttcgccaccgcacacatccacggagagatctacaggcgccggggctggctgacctctgagg
gcaaggagatcaagaacaaggatgagatcctggccctgctgaaggccctgtttctgcccaagcggctgag
catcatccactgtcctggacaccagaagggacactccgccgaggcaaggggcaatcggatggccgacc
aggccgccagaaaggctgctattactgaaactcccgacacttccactctgctgattgaaaactcctcccttc
tggcggctcaaaaagaaccgccgacggcagcgaattcgagtctcccaagaagaagaggaaagtcggc
tctggccctgccgctaagagagtgaagctggactaa

Supplementary Note 10. The sequence of uPEn2.

NLS
Cas9 nuclease
Linker
Reverse transcriptase
Ubiquitin

gagagccgccaccatgaaacggacagccgacggaagcgagttcgagtcaccaaagaagaagcgga
aagtcgacaagaagtacagcatcggcctggacatcggcaccaactctgtgggctgggccgtgatcaccg
acgagtacaaggtgcccagcaagaaattcaaggtgctgggcaacaccgaccggcacagcatcaagaa
gaacctgatcggagccctgctgttcgacagcggcgaaacagccgaggccacccggctgaagagaacc
gccagaagaagatacaccagacggaagaaccggatctgctatctgcaagagatcttcagcaacgagat
ggccaaggtggacgacagcttcttccacagactggaagagtccttcctggtggaagaggataagaagca
cgagcggcaccccatcttcggcaacatcgtggacgaggtggcctaccacgagaagtaccccaccatcta
ccacctgagaaagaaactggtggacagcaccgacaaggccgacctgcggctgatctatctggccctggc
ccacatgatcaagttccggggccacttcctgatcgagggcgacctgaaccccgacaacagcgacgtgga
caagctgttcatccagctggtgcagacctacaaccagctgttcgaggaaaaccccatcaacgccagcggc
gtggacgccaaggccatcctgtctgccagactgagcaagagcagaaagctggaaaatctgatcgcccag
ctgcccggcgagaagaagaatggcctgttcggaaacctgattgccctgagcctgggcctgacccccaactt
caagagcaacttcgacctggccgaggatgccaaactgcagctgagcaaggacacctacgacgacgac
ctggacaacctgctggcccagatcggcgaccagtacgccgacctgtttctggccgccaagaacctgtccg
acgccatcctgctgagcgacatcctgagagtgaacaccgagatcaccaaggcccccctgagcgcctctat
gatcaagagatacgacgagcaccaccaggacctgaccctgctgaaagctctcgtgcggcagcagctgcc
tgagaagtacaaagagattttcttcgaccagagcaagaacggctacgccggctacattgacggcggagc
cagccaggaagagttctacaagttcatcaagcccatcctggaaaagatggacggcaccgaggaactgct
cgtgaagctgaagagagaggacctgctgcggaagcagcggaccttcgacaacggcagcatcccccac
cagatccacctgggagagctgcacgccattctgcggcggcaggaagatttttacccattcctgaaggacaa
ccgggaaaagatcgagaagatcctgaccttccgcatcccctactacgtgggccctctggccaggggaaac
agcagattcgcctggatgaccagaaagagcgaggaaaccatcaccccctggaacttcgaggaagtggt
ggacaagggcgcttccgcccagagcttcatcgagcggatgaccaacttcgataagaacctgcccaacga
gaaggtgctgcccaagcacagcctgctgtacgagtacttcaccgtgtataacgagctgaccaaagtgaaat
acgtgaccgagggaatgagaaagcccgccttcctgagcggcgagcagaaaaaggccatcgtggacctg
ctgttcaagaccaaccggaaagtgaccgtgaagcagctgaaagaggactacttcaagaaaatcgagtgc

ttcgactccgtggaaatctccggcgtggaagatcggttcaacgcctccctgggcacataccacgatctgctg
aaaattatcaaggacaaggacttcctggacaatgaggaaaacgaggacattctggaagatatcgtgctga
ccctgacactgtttgaggacagagagatgatcgaggaacggctgaaaacctatgcccacctgttcgacga
caaagtgatgaagcagctgaagcggcggagatacaccggctggggcaggctgagccggaagctgatc
aacggcatccgggacaagcagtccggcaagacaatcctggatttcctgaagtccgacggcttcgccaac
agaaacttcatgcagctgatccacgacgacagcctgacctttaaagaggacatccagaaagcccaggtgt
ccggccagggcgatagcctgcacgagcacattgccaatctggccggcagccccgccattaagaagggc
atcctgcagacagtgaaggtggtggacgagctcgtgaaagtgatgggccggcacaagcccgagaacat
cgtgatcgaaatggccagagagaaccagaccacccagaagggacagaagaacagccgcgagagaa
tgaagcggatcgaagagggcatcaaagagctgggcagccagatcctgaaagaacaccccgtggaaaa
cacccagctgcagaacgagaagctgtacctgtactacctgcagaatgggcgggatatgtacgtggaccag
gaactggacatcaaccggctgtccgactacgatgtggacgctatcgtgcctcagagctttctgaaggacga
ctccatcgacaacaaggtgctgaccagaagcgacaagaaccggggcaagagcgacaacgtgccctcc
gaagaggtcgtgaagaagatgaagaactactggcggcagctgctgaacgccaagctgattacccagag
aaagttcgacaatctgaccaaggccgagagaggcggcctgagcgaactggataaggccggcttcatca
agagacagctggtggaaacccggcagatcacaaagcacgtggcacagatcctggactcccggatgaac
actaagtacgacgagaatgacaagctgatccgggaagtgaaagtgatcaccctgaagtccaagctggtgt
ccgatttccggaaggatttccagttttacaaagtgcgcgagatcaacaactaccaccacgcccacgacgcc
tacctgaacgccgtcgtgggaaccgccctgatcaaaaagtaccctaagctggaaagcgagttcgtgtacg
gcgactacaaggtgtacgacgtgcggaagatgatcgccaagagcgagcaggaaatcggcaaggctac
cgccaagtacttcttctacagcaacatcatgaactttttcaagaccgagattaccctggccaacggcgagat
ccggaagcggcctctgatcgagacaaacggcgaaaccggggagatcgtgtgggataagggccgggatt
ttgccaccgtgcggaaagtgctgagcatgccccaagtgaatatcgtgaaaaagaccgaggtgcagacag
gcggcttcagcaaagagtctatcctgcccaagaggaacagcgataagctgatcgccagaaagaaggac
tgggaccctaagaagtacggcggcttcgacagccccaccgtggcctattctgtgctggtggtggccaaagt
ggaaaagggcaagtccaagaaactgaagagtgtgaaagagctgctggggatcaccatcatggaaaga
agcagcttcgagaagaatcccatcgactttctggaagccaagggctacaaagaagtgaaaaaggacctg
atcatcaagctgcctaagtactccctgttcgagctggaaaacggccggaagagaatgctggcctctgccgg
cgaactgcagaagggaaacgaactggccctgccctccaaatatgtgaacttcctgtacctggccagccact
atgagaagctgaagggctcccccgaggataatgagcagaaacagctgtttgtggaacagcacaagcact
acctggacgagatcatcgagcagatcagcgagttctccaagagagtgatcctggccgacgctaatctgga
caaagtgctgtccgcctacaacaagcaccgggataagcccatcagagagcaggccgagaatatcatcc
acctgtttaccctgaccaatctgggagcccctgccgccttcaagtactttgacaccaccatcgaccggaaga
ggtacaccagcaccaaagaggtgctggacgccaccctgatccaccagagcatcaccggcctgtacgag
acacggatcgacctgtctcagctgggaggtgactccggcggaagctctggtggcagcaagcggaccgcc
gacggctctgaattcgagagccctaagaagaaaagaaaggtgagcggaggctctagcggcggaagca
ccctgaacattgaagacgagtatagactgcatgaaacaagcaaggaacccgacgtgtccctgggctcca
cctggctgtccgactttccccaggcctgggccgagacaggaggaatgggcctggccgtgcggcaggcac
ccctgatcatccctctgaaggccacctctacacccgtgagcatcaagcagtaccctatgtctcaggaggcc
agactgggcatcaagcctcacatccagaggctgctggaccagggcatcctggtgccatgccagagcccct
ggaacacaccactgctgcccgtgaagaagccaggcaccaatgactatagacccgtgcaggatctgaga
gaggtgaacaagagggtggaggatatccaccccaccgtgcccaacccttacaatctgctgtccggcctgc
cccccttctcaccagtggtatacagtgctggacctgaaggatgccttcttttgtctgagactgcaccctaccagc
cagccactgttcgcctttgagtggagggaccctgagatgggcatctctggccagctgacctggacacgcct

gcctcagggcttcaagaatagcccaacactgtttaacgaggccctgcaccgcgacctggcagatttccgga
tccagcacccagatctgatcctgctgcagtacgtggacgatctgctgctggccgccaccagcgagctggatt
gccagcagggaacacgcgccctgctgcagaccctgggaaacctgggatatagggcatccgccaagaa
ggcccagatctgtcagaagcaggtgaagtacctgggctatctgctgaaggagggccagagatggctgac
agaggccaggaaggagacagtgatgggccagccaacacccaagaccccaagacagctgagggagtt
cctgggcaaagcaggattttgcaggctgttcatcccaggattcgcagagatggcagcacctctgtacccact
gaccaagccgggcaccctgtttaattggggccctgaccagcagaaggcctatcaggagatcaagcaggc
cctgctgacagcaccagccctgggcctgccagacctgaccaagcctttcgagctgtttgtggatgagaagc
agggctacgccaagggcgtgctgacccagaagctgggaccatggagacggcccgtggcctatctgtcca
agaagctggacccagtggcagcaggatggccaccatgcctgaggatggtggcagcaatcgccgtgctga
caaaggatgccggcaagctgaccatgggacagccactggtcatcctggcaccacacgcagtggaggcc
ctggtgaagcagcctccagatcgctggctgtctaacgcccggatgacacactaccaggccctgctgctgga
caccgatcgcgtgcagtttggccctgtggtggccctgaatccagccaccctgctgcctctgccagaggagg
gcctgcagcacaactgtctggacatcctggcagaggcacacggaacaaggccagacctgaccgatcag
cccctgcctgacgccgatcacacatggtataccgatggaagctccctgctgcaggagggccagaggaag
gcaggagcagcagtgaccacagagacagaagtgatctgggccaaggccctgccagcaggcacatccg
cccagcgggccgagctgatcgccctgacccaggccctgaagatggccgagggcaagaagctgaacgt
gtacacagactccagatatgccttcgccaccgcacacatccacggagagatctacaggcgccggggctg
gctgacctctgagggcaaggagatcaagaacaaggatgagatcctggccctgctgaaggccctgtttctgc
ccaagcggctgagcatcatccactgtcctggacaccagaagggacactccgccgaggcaaggggcaat
cggatggccgaccaggccgccagaaaggctgctattactgaaactcccgacacttccactctgctgattga
aaactcctcccccttccggcggaagctctggtggcagcaagcggaccgccgacggctctgaattcgagagc
cctaagaagaaaagaaaggtgagcggaggctctagcggcggaagcgccgccagtttaaacggcgcgc
cattaattaaggatccaatgttgattttcgtgaaaacccttaccgggaaaaccatcaccctcgaggttgaacc
ctcggatacgatagaaaatgtaaaggccaagatccaggataaggaaggaattcctcctgatcagcagag
actggcctttgctggcaaatcgctggaagatggacgtactttgtctgactacaatattctaaaggactctaaac
ttcatcctctgttgagacttcgttctggcggctcaaaaagaaccgccgacggcagcgaattcgagtctccca
agaagaagaggaaagtcggctctggccctgccgctaagagagtgaagctggactaa

Supplementary Note 11. The sequence of uPEn3.

NLS

Cas9 nuclease

Linker

P2A

Reverse transcriptase

Ubiquitin

gagagccgccaccatgaaacggacagccgacggaagcgagttcgagtcaccaaagaagaagcgga
aagtcgacaagaagtacagcatcggcctggacatcggcaccaactctgtgggctgggccgtgatcaccg
acgagtacaaggtgcccagcaagaaattcaaggtgctgggcaacaccgaccggcacagcatcaagaa
gaacctgatcggagccctgctgttcgacagcggcgaaacagccgaggccacccggctgaagagaacc
gccagaagaagatacaccagacggaagaaccggatctgctatctgcaagagatcttcagcaacgagat
ggccaaggtggacgacagcttcttccacagactggaagagtccttcctggtggaagaggataagaagca
cgagcggcaccccatcttcggcaacatcgtggacgaggtggcctaccacgagaagtaccccaccatcta
ccacctgagaaagaaactggtggacagcaccgacaaggccgacctgcggctgatctatctggccctggc

ccacatgatcaagttccggggccacttcctgatcgagggcgacctgaaccccgacaacagcgacgtgga
caagctgttcatccagctggtgcagacctacaaccagctgttcgaggaaaaccccatcaacgccagcggc
gtggacgccaaggccatcctgtctgccagactgagcaagagcagaaagctggaaaatctgatcgcccag
ctgcccggcgagaagaagaatggcctgttcggaaacctgattgccctgagcctgggcctgacccccaactt
caagagcaacttcgacctggccgaggatgccaaactgcagctgagcaaggacacctacgacgacgac
ctggacaacctgctggcccagatcggcgaccagtacgccgacctgtttctggccgccaagaacctgtccg
acgccatcctgctgagcgacatcctgagagtgaacaccgagatcaccaaggcccccctgagcgcctctat
gatcaagagatacgacgagcaccaccaggacctgaccctgctgaaagctctcgtgcggcagcagctgcc
tgagaagtacaaagagattttcttcgaccagagcaagaacggctacgccggctacattgacggcggagc
cagccaggaagagttctacaagttcatcaagcccatcctggaaaagatggacggcaccgaggaactgct
cgtgaagctgaagagagaggacctgctgcggaagcagcggaccttcgacaacggcagcatcccccac
cagatccacctgggagagctgcacgccattctgcggcggcaggaagatttttacccattcctgaaggacaa
ccgggaaaagatcgagaagatcctgaccttccgcatcccctactacgtgggccctctggccaggggaaac
agcagattcgcctggatgaccagaaagagcgaggaaaccatcaccccctggaacttcgaggaagtggt
ggacaagggcgcttccgcccagagcttcatcgagcggatgaccaacttcgataagaacctgcccaacga
gaaggtgctgcccaagcacagcctgctgtacgagtacttcaccgtgtataacgagctgaccaaagtgaaat
acgtgaccgagggaatgagaaagcccgccttcctgagcggcgagcagaaaaaggccatcgtggacctg
ctgttcaagaccaaccggaaagtgaccgtgaagcagctgaaagaggactacttcaagaaaatcgagtgc
ttcgactccgtggaaatctccggcgtggaagatcggttcaacgcctccctgggcacataccacgatctgctg
aaaattatcaaggacaaggacttcctggacaatgaggaaaacgaggacattctggaagatatcgtgctga
ccctgacactgtttgaggacagagagatgatcgaggaacggctgaaaacctatgcccacctgttcgacga
caaagtgatgaagcagctgaagcggcggagatacaccggctggggcaggctgagccggaagctgatc
aacggcatccgggacaagcagtccggcaagacaatcctggatttcctgaagtccgacggcttcgccaac
agaaacttcatgcagctgatccacgacgacagcctgacctttaaagaggacatccagaaagcccaggtgt
ccggccagggcgatagcctgcacgagcacattgccaatctggccggcagccccgccattaagaagggc
atcctgcagacagtgaaggtggtggacgagctcgtgaaagtgatgggccggcacaagcccgagaacat
cgtgatcgaaatggccagagagaaccagaccacccagaagggacagaagaacagccgcgagagaa
tgaagcggatcgaagagggcatcaaagagctgggcagccagatcctgaaagaacaccccgtggaaaa
cacccagctgcagaacgagaagctgtacctgtactacctgcagaatgggcgggatatgtacgtggaccag
gaactggacatcaaccggctgtccgactacgatgtggacgctatcgtgcctcagagctttctgaaggacga
ctccatcgacaacaaggtgctgaccagaagcgacaagaaccggggcaagagcgacaacgtgccctcc
gaagaggtcgtgaagaagatgaagaactactggcggcagctgctgaacgccaagctgattacccagag
aaagttcgacaatctgaccaaggccgagagaggcggcctgagcgaactggataaggccggcttcatca
agagacagctggtggaaacccggcagatcacaaagcacgtggcacagatcctggactcccggatgaac
actaagtacgacgagaatgacaagctgatccgggaagtgaaagtgatcaccctgaagtccaagctggtgt
ccgatttccggaaggatttccagttttacaaagtgcgcgagatcaacaactaccaccacgcccacgacgcc
tacctgaacgccgtcgtgggaaccgccctgatcaaaaagtaccctaagctggaaagcgagttcgtgtacg
gcgactacaaggtgtacgacgtgcggaagatgatcgccaagagcgagcaggaaatcggcaaggctac
cgccaagtacttcttctacagcaacatcatgaactttttcaagaccgagattaccctggccaacggcgagat
ccggaagcggcctctgatcgagacaaacggcgaaaccggggagatcgtgtgggataagggccgggatt
ttgccaccgtgcggaaagtgctgagcatgccccaagtgaatatcgtgaaaaagaccgaggtgcagacag
gcggcttcagcaaagagtctatcctgcccaagaggaacagcgataagctgatcgccagaaagaaggac
tgggaccctaagaagtacggcggcttcgacagccccaccgtggcctattctgtgctggtggtggccaaagt
ggaaaagggcaagtccaagaaactgaagagtgtgaaagagctgctggggatcaccatcatggaaaga

agcagcttcgagaagaatcccatcgactttctggaagccaagggctacaaagaagtgaaaaaggacctg
atcatcaagctgcctaagtactccctgttcgagctggaaaacggccggaagagaatgctggcctctgccgg
cgaactgcagaagggaaacgaactggccctgccctccaaatatgtgaacttcctgtacctggccagccact
atgagaagctgaagggctcccccgaggataatgagcagaaacagctgtttgtggaacagcacaagcact
acctggacgagatcatcgagcagatcagcgagttctccaagagagtgatcctggccgacgctaatctgga
caaagtgctgtccgcctacaacaagcaccgggataagcccatcagagagcaggccgagaatatcatcc
acctgtttaccctgaccaatctgggagcccctgccgccttcaagtactttgacaccaccatcgaccggaaga
ggtacaccagcaccaaagaggtgctggacgccaccctgatccaccagagcatcaccggcctgtacgag
acacggatcgacctgtctcagctgggaggtgactccggcggaagctctggtggcagcaagcggaccgcc
gacggctctgaattcgagagccctaagaagaaaagaaaggtgagcggaggctctagcggcggaagca
ccctgaacattgaagacgagtatagactgcatgaaacaagcaaggaacccgacgtgtccctgggctcca
cctggctgtccgactttccccaggcctgggccgagacaggaggaatgggcctggccgtgcggcaggcac
ccctgatcatccctctgaaggccacctctacacccgtgagcatcaagcagtaccctatgtctcaggaggcc
agactgggcatcaagcctcacatccagaggctgctggaccagggcatcctggtgccatgccagagcccct
ggaacacaccactgctgcccgtgaagaagccaggcaccaatgactatagacccgtgcaggatctgaga
gaggtgaacaagagggtggaggatatccaccccaccgtgcccaacccttacaatctgctgtccggcctgc
cccctctcaccagtggtatacagtgctggacctgaaggatgccttcttttgtctgagactgcaccctaccagc
cagccactgttcgcctttgagtggagggaccctgagatgggcatctctggccagctgacctggacacgcct
gcctcagggcttcaagaatagcccaacactgtttaacgaggccctgcaccgcgacctggcagatttccgga
tccagcacccagatctgatcctgctgcagtacgtggacgatctgctgctggccgccaccagcgagctggatt
gccagcagggaacacgcgccctgctgcagaccctgggaaacctgggatatagggcatccgccaagaa
ggcccagatctgtcagaagcaggtgaagtacctgggctatctgctgaaggagggccagagatggctgac
agaggccaggaaggagacagtgatgggccagccaacacccaagaccccaagacagctgagggagtt
cctgggcaaagcaggattttgcaggctgttcatcccaggattcgcagagatggcagcacctctgtacccact
gaccaagccgggcaccctgtttaattggggccctgaccagcagaaggcctatcaggagatcaagcaggc
cctgctgacagcaccagccctgggcctgccagacctgaccaagcctttcgagctgtttgtggatgagaagc
agggctacgccaagggcgtgctgacccagaagctgggaccatggagacggcccgtggcctatctgtcca
agaagctggacccagtggcagcaggatggccaccatgcctgaggatggtggcagcaatcgccgtgctga
caaaggatgccggcaagctgaccatgggacagccactggtcatcctggcaccacacgcagtggaggcc
ctggtgaagcagcctccagatcgctggctgtctaacgcccggatgacacactaccaggccctgctgctgga
caccgatcgcgtgcagtttggccctgtggtggccctgaatccagccaccctgctgcctctgccagaggagg
gcctgcagcacaactgtctggacatcctggcagaggcacacggaacaaggccagacctgaccgatcag
cccctgcctgacgccgatcacacatggtataccgatggaagctccctgctgcaggagggccagaggaag
gcaggagcagcagtgaccacagagacagaagtgatctgggccaaggccctgccagcaggcacatccg
cccagcgggccgagctgatcgccctgacccaggccctgaagatggccgagggcaagaagctgaacgt
gtacacagactccagatatgccttcgccaccgcacacatccacggagagatctacaggcgccggggctg
gctgacctctgagggcaaggagatcaagaacaaggatgagatcctggccctgctgaaggccctgtttctgc
ccaagcggctgagcatcatccactgtcctggacaccagaagggacactccgccgaggcaaggggcaat
cggatggccgaccaggccgccagaaaggctgctattactgaaactcccgacacttccactctgctgattga
aaactcctcccctctggcggctcaaaaagaaccgccgacggcagcgaattcgagtctcccaagaagaa
gaggaaagtcggctctggccctgccgctaagagagtgaagctggacggatccggcgcaacaaacttctct
ctgctgaaacaagccggagatgtcgaagagaatcctggaccgatgcatatgaaacggacagccgacgg
aagcgagttcgagtcaccaaagaagaagcggaaagtcgccgccagtttaaacggcgcgccattaattaa
ggatccaatgttgattttcgtgaaaacccttaccgggaaaaccatcaccctcgaggttgaaccctcggatac

gatagaaaatgtaaaggccaagatccaggataaggaaggaattcctcctgatcagcagagactggcctttgctggcaaatcgctggaagatggacgtactttgtctgactacaatattctaaaggactctaaacttcatcctctgttgagacttcgttaa

Original scanned blots for Western results presented in the supplementary figures

In Suppl Fig. 4f

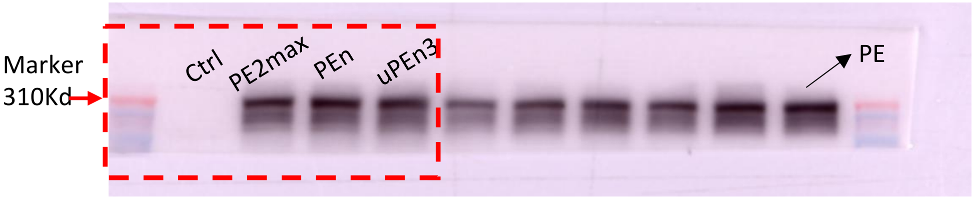

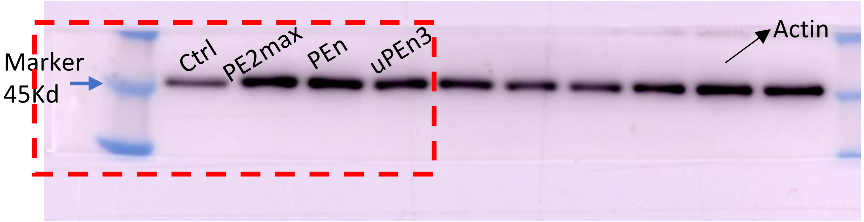

In Suppl Fig. 6b

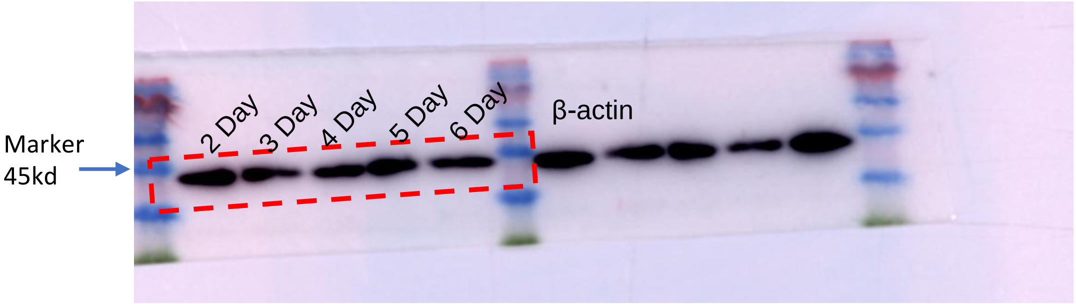

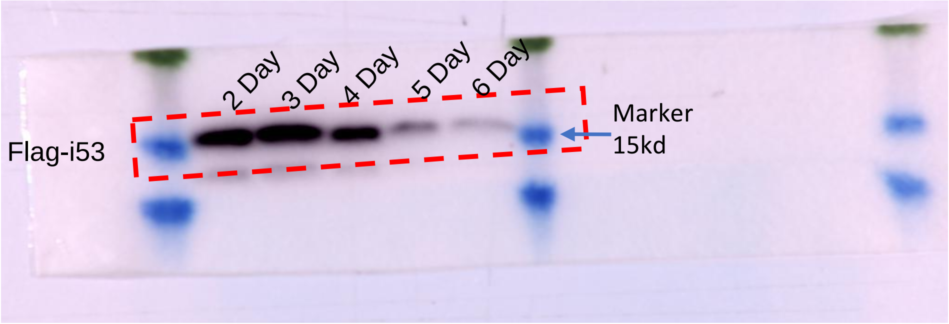

Supplement: Supplementary file 1 — Supplementary Information [file 41467_2023_35870_MOESM1_ESM.pdf]
