# Supplementary material for: Development of a versatile nuclease prime editor with upgraded precision
Source: Nat Commun. 2023 Jan 19;14:305. doi: 10.1038/s41467-023-35870-0 (PMC9852468; doi:10.1038/s41467-023-35870-0)
Supplement: Supplementary file 2 — Reporting Summary [file 41467_2023_35870_MOESM2_ESM.pdf]

nature portfolio

Corresponding author(s): Xingxu Huang

Last updated by author(s): Dec 28, 2022

Reporting Summary

Nature Portfolio wishes to improve the reproducibility of the work that we publish. This form provides structure for consistency and transparency in reporting. For further information on Nature Portfolio policies, see our Editorial Policies and the Editorial Policy Checklist.

Statistics

For all statistical analyses, confirm that the following items are present in the figure legend, table legend, main text, or Methods section.

n/a	Confirmed	
☐	☒	The exact sample size (*n*) for each experimental group/condition, given as a discrete number and unit of measurement
☐	☒	A statement on whether measurements were taken from distinct samples or whether the same sample was measured repeatedly
☐	☒	The statistical test(s) used AND whether they are one- or two-sided<br>*Only common tests should be described solely by name; describe more complex techniques in the Methods section.*
☒	☐	A description of all covariates tested
☒	☐	A description of any assumptions or corrections, such as tests of normality and adjustment for multiple comparisons
☐	☒	A full description of the statistical parameters including central tendency (e.g. means) or other basic estimates (e.g. regression coefficient) AND variation (e.g. standard deviation) or associated estimates of uncertainty (e.g. confidence intervals)
☐	☒	For null hypothesis testing, the test statistic (e.g. *F*, *t*, *r*) with confidence intervals, effect sizes, degrees of freedom and *P* value noted<br>*Give P values as exact values whenever suitable.*
☒	☐	For Bayesian analysis, information on the choice of priors and Markov chain Monte Carlo settings
☒	☐	For hierarchical and complex designs, identification of the appropriate level for tests and full reporting of outcomes
☒	☐	Estimates of effect sizes (e.g. Cohen's *d*, Pearson's *r*), indicating how they were calculated

Our web collection on statistics for biologists contains articles on many of the points above.

Software and code

Policy information about availability of computer code

Data collection	Illumina Nova-seq6000 platform (2x PE150 and 2x PE250), FACSDiva (8.0.1)
Data analysis	Cas-OFFinder (v2.4), CRISPResso2(v2.2.7), Graphpad prism (v.9.1.1).<br>For deep sequencing analyses, the sequencing reads were demultiplexed using AdapterRemoval (version 2.2.2), and the pair-end reads with 11 bp or more alignments were combined into a single consensus read. All processed reads were then mapped to the target sequences using the BWA-MEM algorithm (BWA v0.7.17). For targeted insertions, deletions, replacements, prime editing efficiency was calculated as: Accurate edits: [number of reads with the desired edit that do not contain indels]/total mapped reads (%); All RT-driven edits: [number of reads with the desired edit and imperfect edit that contain extra RT template sequence]/total mapped reads (%); Indel frequency was calculated as: number of indel-containing reads/total mapped reads (%). For base conversion, prime editing efficiency was calculated as: [number of reads with the desired edit that do not contain indels]/total mapped reads (%); Indel frequency was calculated as: number of indel-containing reads/total mapped reads (%).<br>For some experiments, the demultiplexed pair-ended reads were subjected to analyses by CRISPResso2 under the HDR mode to generate the explicit demonstrations of allele distributions. The indel quantification windows were set to extend 30-bp beyond the positions of the cleavage site and the end position of the template-dependent RT. For sequence alignment, the plot_window_size was adjusted according to the combined sizes of the insertion and the HR.<br>For WGS analyses, all cleaned reads were mapped to the human reference genome (GRCh38/hg38) using BWA v.0.7.17 with default parameters. Sequence reads were removed for duplicates using Sambamba v.0.6.7. Variants were identified by GATK (v.4.1.8.1) HaplotypeCaller and filtered with the following criteria: (1) sequencing depth (for each individual) > 1/3× and < 3×; (2) variant confidence/quality by depth > 2; (3) RMS mapping quality (MQ) > 40.0; (4) Phred-scaled P value using Fisher's exact test to detect strand bias < 60; (5) Z-score from the Wilcoxon rank sum test of Alt vs. Ref read MQs (MQRankSum) > −12.5; and (6) Z-score from the Wilcoxon rank sum test of Alt vs. Ref read position bias (ReadPosRankSum) > −8. Reference sequencing data from un-transfected cells were used to filter pre-existing

mutations. Potential off-target sites were predicted by Cas-OFFinder.

For manuscripts utilizing custom algorithms or software that are central to the research but not yet described in published literature, software must be made available to editors and reviewers. We strongly encourage code deposition in a community repository (e.g. GitHub). See the Nature Portfolio guidelines for submitting code & software for further information.

Data

Policy information about availability of data

All manuscripts must include a data availability statement. This statement should provide the following information, where applicable:

- Accession codes, unique identifiers, or web links for publicly available datasets
- A description of any restrictions on data availability
- For clinical datasets or third party data, please ensure that the statement adheres to our policy

Targeted amplicon sequencing and WGS data have been deposited to the NCBI-SRA repository under BioProject number: PRJNA847383 (https://www.ncbi.nlm.nih.gov/bioproject/PRJNA847383/). Descriptions of the treatments and samples included in the dataset are provided in Supplementary Data 1 (in a sheet named "SRA"). The reference human genome assembly GRCh38/hg38 used for reads mapping is an openly accessible resource (https://www.ncbi.nlm.nih.gov/assembly/GCF_000001405.40). Source data are provided with this paper.

Human research participants

Policy information about studies involving human research participants and Sex and Gender in Research.

Reporting on sex and gender	none
Population characteristics	none
Recruitment	none
Ethics oversight	none

Note that full information on the approval of the study protocol must also be provided in the manuscript.

Field-specific reporting

Please select the one below that is the best fit for your research. If you are not sure, read the appropriate sections before making your selection.

[x] Life sciences [ ] Behavioural & social sciences [ ] Ecological, evolutionary & environmental sciences

For a reference copy of the document with all sections, see nature.com/documents/nr-reporting-summary-flat.pdf

Life sciences study design

All studies must disclose on these points even when the disclosure is negative.

Sample size	Samples sizes were indicated in figure legends. We did not predetermine sample sizes. The sample sizes for our experiments were based on related studies in the field (see Refs: Anzalone A, Randolph P, Davis J et al., 2019 [10.1038/s41586-019-1711-4]; Liu P, Liang S, Zheng C et al., 2021 [10.1038/s41467-021-22295-w]; Lin Q, Jin S, Zong Y et al., 2021 [10.1038/s41587-021-00868-w]; Nelson J, Randolph P, Shen S et al., 2021 [10.1038/s41587-021-01039-7]).
Data exclusions	No data has been excluded from the analyses.
Replication	As indicated in the figure legends, all data represented are results from biological replicates. The average values from such replications are presented (+/- STD, as indicated in the legend). In addition, the conclusions are made on analyses at multiple target sites.
Randomization	No randomization of samples was applied. In any given experiment within this study, a same mammalian cell line was used and the cells were cultured under the same condition.
Blinding	Blinding was not applied. For most of the experiments, a series of samples representing different groups are analyzed side-by-side in a single trial. All measurements in this study are highly objective (deep sequencing, chemiluminescence analyses, etc).

Reporting for specific materials, systems and methods

We require information from authors about some types of materials, experimental systems and methods used in many studies. Here, indicate whether each material, system or method listed is relevant to your study. If you are not sure if a list item applies to your research, read the appropriate section before selecting a response.

Materials & experimental systems

n/a	Involved in the study
☐	☒ Antibodies
☐	☒ Eukaryotic cell lines
☒	☐ Palaeontology and archaeology
☒	☐ Animals and other organisms
☒	☐ Clinical data
☒	☐ Dual use research of concern

Methods

n/a	Involved in the study
☒	☐ ChIP-seq
☐	☒ Flow cytometry
☒	☐ MRI-based neuroimaging

Antibodies

Antibodies used	anti-Cas9 (Abcam ab204448), anti-β-actin (Absin abs132001), or anti-Flag (Abcam ab205606) antibodies
Validation	The anti-Cas9 antibody has been characterized by the vendor (https://www.abcam.cn/crispr-cas9-antibody-ab204448.html) for ICC/IF, IP, Flow Cyt, WB.<br>The anti-β-actin antibody is validated by the vendor (https://www.absin.cn/beta-actin-antibody/abs132001.html) for WB , IHC 1:200, IF/ICC , ELISA.<br>The anti-Flag antibody is validated by the vendor (https://www.abcam.cn/ddddk-tag-binds-to-flag-tag-sequence-antibody-epr20018-251-ab205606.html) for WB, ICC/IF, Flow Cyt, IHC-P, IP.

Eukaryotic cell lines

Policy information about cell lines and Sex and Gender in Research

Cell line source(s)	HEK293T (ATCC CRL-3216), U2OS (ATCC HTB-96) and HeLa (ATCC CCL-2) cells
Authentication	HEK293T was not authenticated. Other cell lines were authenticated by the supplier by STR typing.
Mycoplasma contamination	All cell lines were tested negative of mycoplasma contaminations.
Commonly misidentified lines<br>(See ICLAC register)	None of the cell lines used are listed in the ICLAC database.

Flow Cytometry

Plots

Confirm that:

☒ The axis labels state the marker and fluorochrome used (e.g. CD4-FITC).

☒ The axis scales are clearly visible. Include numbers along axes only for bottom left plot of group (a 'group' is an analysis of identical markers).

☒ All plots are contour plots with outliers or pseudocolor plots.

☒ A numerical value for number of cells or percentage (with statistics) is provided.

Methodology

Sample preparation	The transfected cells were trypsinized for preparation of single-cell suspensions. After washing with PBS, the cells were subjected to flow cytometry analyses
Instrument	BD AriaII for sorting
Software	FACSDiva (8.0.1) for collections
Cell population abundance	Cells were harvested with EGFP+ (or EGFP+/mCherry+) selection, a total of around 10,000 cells were sorted for subsequent genomic DNA preparetion.
Gating strategy	EGFP+ gate or EGFP+/mCherry+ gate was respectively used for sorting, and both gates with the same range of FL1-fluorescence.

☒ Tick this box to confirm that a figure exemplifying the gating strategy is provided in the Supplementary Information.
