# Supplementary material for: Development of a versatile nuclease prime editor with upgraded precision
Source: Nat Commun. 2023 Jan 19;14:305. doi: 10.1038/s41467-023-35870-0 (PMC9852468; doi:10.1038/s41467-023-35870-0)
Supplement: Supplementary file 3 — Description of Additional Supplementary Files [file 41467_2023_35870_MOESM3_ESM.pdf]

Title: Supplementary Data 1

Description: Sequences for HRpegRNAs, sequences for PE-adapted pegRNAs, primers for target amplification, primers for offtarget site amplification, and descriptions for samples and experiments associated with uploaded dataset at SRA
